# Structural insights into the covalent regulation of PAPP-A activity by proMBP and STC2

**DOI:** 10.1038/s41421-022-00502-2

**Published:** 2022-12-22

**Authors:** Qihang Zhong, Honglei Chu, Guopeng Wang, Cheng Zhang, Rong Li, Fusheng Guo, Xinlu Meng, Xiaoguang Lei, Youli Zhou, Ruobing Ren, Lin Tao, Ningning Li, Ning Gao, Yuan Wei, Jie Qiao, Jing Hang

**Affiliations:** 1 Center for Reproductive Medicine, Department of Obstetrics and Gynecology, Peking University Third Hospital, Beijing, China; 2 Key Laboratory of Assisted Reproduction, Ministry of Education, Beijing, China; 3 Beijing Key Laboratory of Reproductive Endocrinology and Assisted Reproduction, Beijing, China; 4 National Clinical Research Center for Obstetrics and Gynecology, Beijing, China; 5 State Key Laboratory of Membrane Biology, School of Life Sciences, Peking University, Beijing, China; 6 Department of Gastrointestinal Oncology, Key Laboratory of Carcinogenesis and Translational Research (Ministry of Education/Beijing), Peking University Cancer Hospital & Institute, Beijing, China; 7 Beijing National Laboratory for Molecular Sciences, State Key Laboratory of Natural and Biomimetic Drugs, Key Laboratory of Bioorganic Chemistry and Molecular Engineering of Ministry of Education, Department of Chemical Biology, College of Chemistry and Molecular Engineering, Synthetic and Functional Biomolecules Center, Peking University, Beijing, China; 8 Peking-Tsinghua Center for Life Sciences, Peking University, Beijing, China; 9 Institute for Cancer Research, Shenzhen Bay Laboratory, Shenzhen, Guangdong China; 10 School of Life and Health Sciences, The Chinese University of Hong Kong, Shenzhen, Guangdong China; 11 Shanghai Key Laboratory of Metabolic Remodeling and Health, Institute of Metabolism and Integrative Biology, Fudan University, Shanghai, China; 12 Department of Orthopedics, First Hospital of China Medical University, Shenyang, Liaoning China

**Keywords:** Cryoelectron microscopy, Proteolysis

## Abstract

Originally discovered in the circulation of pregnant women as a protein secreted by placental trophoblasts, the metalloprotease pregnancy-associated plasma protein A (PAPP-A) is also widely expressed by many other tissues. It cleaves insulin-like growth factor-binding proteins (IGFBPs) to increase the bioavailability of IGFs and plays essential roles in multiple growth-promoting processes. While the vast majority of the circulatory PAPP-A in pregnancy is proteolytically inactive due to covalent inhibition by proform of eosinophil major basic protein (proMBP), the activity of PAPP-A can also be covalently inhibited by another less characterized modulator, stanniocalcin-2 (STC2). However, the structural basis of PAPP-A proteolysis and the mechanistic differences between these two modulators are poorly understood. Here we present two cryo-EM structures of endogenous purified PAPP-A in complex with either proMBP or STC2. Both modulators form 2:2 heterotetramer with PAPP-A and establish extensive interactions with multiple domains of PAPP-A that are distal to the catalytic cleft. This exosite-binding property results in a steric hindrance to prevent the binding and cleavage of IGFBPs, while the IGFBP linker region-derived peptides harboring the cleavage sites are no longer sensitive to the modulator treatment. Functional investigation into proMBP-mediated PAPP-A regulation in selective intrauterine growth restriction (sIUGR) pregnancy elucidates that PAPP-A and proMBP collaboratively regulate extravillous trophoblast invasion and the consequent fetal growth. Collectively, our work reveals a novel covalent exosite-competitive inhibition mechanism of PAPP-A and its regulatory effect on placental function.

## Introduction

Insulin-like growth factors (IGFs) play pleiotropic roles in cellular growth and its bioactivity could be fine-tuned by the direct binding of IGF-binding proteins (IGFBPs)^1,2^. Pregnancy-associated plasma protein A (PAPP-A) is a zinc metalloprotease that explicitly cleaves IGFBPs (primarily IGFBP4 as well as IGFBP2 and IGFBP5), thereby enhancing the local bioavailability of IGFs and promoting cell growth^3^. The proteolytic activity of PAPP-A could be inhibited by several modulators including proform of eosinophil major basic protein (proMBP)^4^, stanniocalcin-1 (STC1)^5^, and stanniocalcin-2 (STC2)^6^. Therefore, the modulator → PAPP-A → IGFBPs → IGFs signaling cascade constitutes an elaborate regulatory axis in promoting embryonic development, osteogenesis, and metabolism, while the aberrance of this axis could result in various pathological consequences including tumor metastasis, neurodegeneration, and aging^7–9^.

PAPP-A belongs to the pappalysin family and its catalytic domain shares only 25%–30% sequence identity with its homologous archetypes present in microorganisms^10^, while pappalysin structures are currently known only for the archaeal ulilysin and the bacterial mirolysin^11,12^. The middle tertile region of PAPP-A displays no global sequence similarity to any other metzincins, and its functions remain enigmatic^13^. In human gestation, placental trophoblasts produce prodigious PAPP-A and then release it into the circulation of both mother and fetus^14^. The concentration of PAPP-A in maternal blood accumulates more than hundreds-fold during the gestation process^15^ but in pathological pregnancies this elevation is dramatically reduced^16^. Accordingly, the concentration of PAPP-A in the first trimester has been widely used as a clinical biomarker in screening for Down syndrome and other pregnancy complications^17,18^.

Interestingly, the vast majority of circulating PAPP-A in pregnancy is inactive and exists as a ~500 kDa disulfide-linked 2:2 heterotetramer with proMBP^19,20^. ProMBP is encoded by *PRG2*, whose aberrant expression is closely related to adverse pregnancy outcomes^21^. *PRG2* is one of the most highly expressed genes in placental extravillous trophoblasts (EVTs)^22^, a differentiated type of placental trophoblasts that migrate deeply into decidual tissue and remodel the maternal spiral arteries, assuring the anchoring of the fetus and the blood supply at the maternal–fetal interface^23^. The dysfunction of EVTs would lead to pregnancy complications^24^ such as selective intrauterine growth restriction (sIUGR), in which one of two monochorionic twins becomes malnourished. Therefore, sIUGR is always widely used for the investigation of trophoblast function^25,26^. Other than proMBP, STC2 is another physiological modulator of PAPP-A by forming a covalent complex with PAPP-A^6^. STC2 serves as a glycosylated hormone that exerts multiple physiopathologic functions especially in tumor progression^27–29^.

Nevertheless, the molecular details concerning the differential interactions between PAPP-A and proMBP or STC2 are unclear. Additionally, the inhibition impact of proMBP towards PAPP-A on placental function and intrauterine fetal growth remains to be elucidated. In this study, we report the cryo-electron microscopy (cryo-EM) structures of PAPP-A·proMBP and PAPP-A·STC2 complexes by utilizing endogenous protein purification and depict the differential modes of inhibition in these two complexes. We also highlight the regulatory effect of PAPP-A activity on placental function. The complex structures together with the biological studies provide that the modulation of the IGF system by PAPP-A and proMBP could regulate EVT invasion and placental function, resulting in the imbalance of twin fetuses in sIUGR, therefore indicating the potential of PAPP-A modulation in clinical applications.

## Results

### Cryo-EM structural determination of the pregnant plasma-purified PAPP-A·proMBP complex

**Fig. 1:**
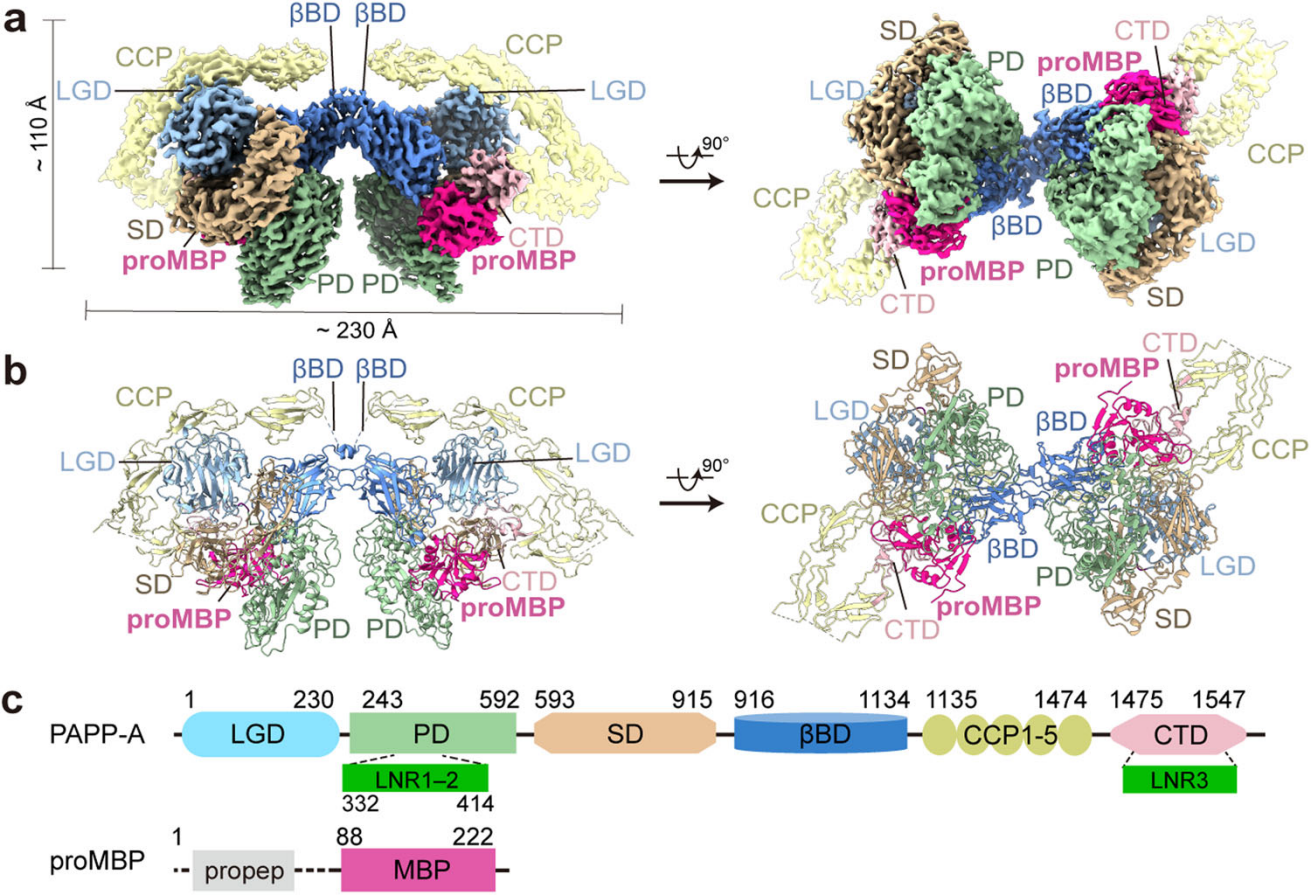
Cryo-EM structure determination of the PAPP-A·proMBP complex. **a** The overall EM density map of the PAPP-A·proMBP complex with a rotation of 90°. **b** The corresponding cartoon representation as in **a**. **c** Schematic domain organization for PAPP-A and proMBP proteins with domain boundaries defined in this study. LGD laminin-G like domain, PD proteolytic domain, LNR lin-12/Notch repeat, SD scarf domain, βBD β-barrel domain, CCP complement control protein, CTD C-terminal domain, propep pro-peptide, MBP matured form of major basic protein. The EM map and the structural figures were generated in either ChimeraX (www.cgl.ucsf.edu/chimeraX/) or PyMOL (www.pymol.org) with the same color scheme applied to all figures.

To assess the molecular mechanism(s) subserving PAPP-A activity and the modulation of proMBP, we purified endogenous PAPP-A·proMBP complex from pregnant human plasma and determined its cryo-EM structure. A combination of several ion-exchange and size-exclusion chromatographic separations was applied to isolate the complex (Supplementary Fig. S1a–d). The presence of full-length proMBP was confirmed by western blotting using a proMBP-specific antibody (Supplementary Fig. S1e) and non-reduced gels showed a major heterotetrameric form for PAPP-A·proMBP (Supplementary Fig. S1f). The cryo-EM analysis generated an initial dimeric 3D map at 3.64 Å resolution, and C2-symmetry expansion was subsequently applied to obtain a final 3.45-Å monomeric half map, which enabled de novo modeling of most of the PAPP-A residues (Supplementary Figs. S1g, h, S2 and Table S1). The dimer cryo-EM map contains two copies of the full-length mature PAPP-A and two copies of proMBP, exhibiting an overall butterfly-shaped structure with a dimension of 230 Å × 110 Å × 90 Å (Fig. 1a). Considering the previous data that lin-12/Notch repeat (LNR) modules act *in trans* within the PAPP-A dimer^30^, we modeled the PAPP-A·proMBP complex as a *trans*-crossed architecture (Fig. 1b; Supplementary Fig. S3a) and therefore the monomeric half model contains PAPP-A domains from two subunits (for simplicity, we used this half model in our manuscript hereafter). The proMBP subunit is tightly held by PAPP-A through multiple contacts, and only the mature region (residues 88–222) was traceable in the EM-density (Fig. 1a, c; Supplementary Video S1).

Based on the structure, we constructed a modified version of the schematic map for the multi-domain organization of PAPP-A: a laminin globular-like jellyroll fold domain (LGD, Arg1–Phe230), a proteolytic catalytic domain (PD, Leu243–Pro592) that contains two LNR modules^31^ (LNR1 and LNR2, Asn332–Tyr414), a crooked scarf domain (SD, Gly593–Gly915), a β-barrel domain (βBD, Asp916–Asp1134), a complement control protein domain (CCP, Cys1135–Cys1474) that contains five modules, and a C-terminal domain (CTD, Val1475–Gly1547) that contains the third LNR module (Fig. 1c). The central region between the PD and the CCP domain that named M1/M2 was previously poorly defined^3^, but in our structure, this region was unambiguously assigned and modeled as SD and βBD (Supplementary Fig. S3b–d). The density at the interface between proMBP and PAPP-A was sufficiently resolved to analyze the detailed interaction (Supplementary Fig. S3e). Besides, plenty of glycosylation sites could be unambiguously assigned (Supplementary Fig. S3f), and a few coordinated Ca^2+^ ions could be identified (Supplementary Fig. S3g). The structure not only confirmed intramolecular disulfide bonds of PAPP-A reported in previous studies^32^, but also identified several new disulfide pairs (Supplementary Fig. S3h, i).

### The overall structure and the intramolecular interactions of PAPP-A

**Fig. 2:**
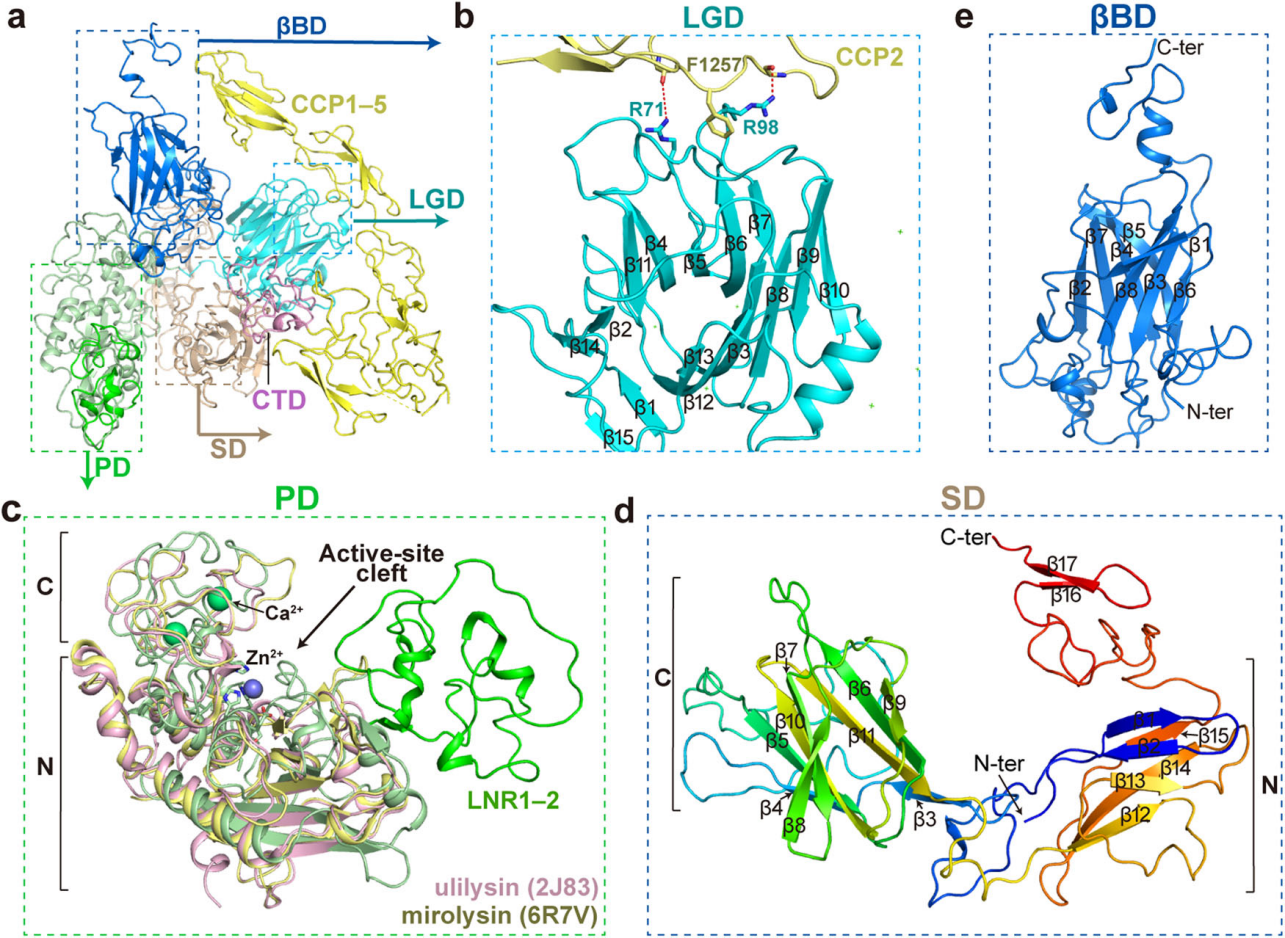
PAPP-A domain organization and the intramolecular interactions. **a** Color-coded overall structure of PAPP-A. Detailed features are zoomed-in in **b**–**e**. **b** The LGD contains fifteen β-strands. The hydrogen bonds between the side chains of Arg71 and Arg98 of the LGD and the main-chain hydroxyl groups of CCP2 are colored red. Phe1257 inserts into a hydrophobic pocket of LGD. **c** The PD is superimposed to its homologs ulilysin (PDB code: 2J83) and mirolysin (PDB code: 6R7V). The Zn^2+^ ion at the active site and the Ca^2+^ ions are shown in blue and green spheres, respectively. **d** The SD composed of 17 β-strands is shown in rainbow color from N-terminus to C-terminus as running from blue to red, respectively. **e** The βBD contains two β-sheets composed of eight β-strands and three small helices.

We first analyzed the overall architecture of PAPP-A using the half model (LGD, PD, SD, and βBD from one subunit and CCP and CTD from the other one) (Fig. 2a). The N-terminal LGD and the catalytic PD locate at both sides of SD, with a linker segment crossing through the SD, and the SD is embedded in the middle to serve as an assembly platform (Supplementary Fig. S4a). LGD consists of two antiparallel β-sheets with seven and eight strands, respectively, and interacts with CCP2 through the intervening loops (Fig. 2b). The side chains of Arg71 and Arg98 in LGD form hydrogen bonds with the main chain hydroxyl groups of CCP2, and the benzene ring of Phe1257 inserts into the hydrophobic cavity in LGD (Fig. 2b).

The catalytic PD could be superimposed onto its pappalysin homologs ulilysin and mirolysin with root-mean-square deviation (RMSD) values of 8.42 Å over 167 Cα atoms and 4.82 Å over 117 Cα atoms, respectively (Fig. 2c). The PD could also be divided into two subdomains: an N-terminal subdomain rich in secondary structure and a C-terminal subdomain mainly comprised of loops (Supplementary Fig. S4b). The active-site cleft is composed of an elongated zinc-finger motif and a functionally important Zn^2+^ ion (Supplementary Fig. S4c). A unique feature of the PAPP-A PD domain, when compared with other metzincins, is the existence of LNR1–2 modules inserted in the N-terminal subdomain which could be unambiguously assigned in the density (Supplementary Fig. S4d). The SD exhibits poor sequence identity to other proteins; based on our structure, it forms a notched dugout architecture and acts as a scaffold for the binding of PD and LGD on two sides. Seventeen β-strands comprise four antiparallel β-sheets and the first two of these are stacked into an N-subdomain and the latter two into C-subdomain (Fig. 2d).

Consisting of two antiparallel β-sheets, βBD locates at the central hub of the structure and links the main body to the following CCP wing (Fig. 2a, e). Due to the inherent flexibility of the five tandem CCP (CCP1–5) modules, only the first two are resolved at side-chain resolution. In combination with the predicted model for CCP3–5 by AlphaFold2^33^, we generated a complete model for CCP1–5. There is an almost 180° bent between CCP4 and CCP5, resulting in the re-orientation of CTD for the binding to proMBP (Supplementary Fig. S4e).

### Cryo-EM structure of the PAPP-A·STC2 complex

**Fig. 3:**
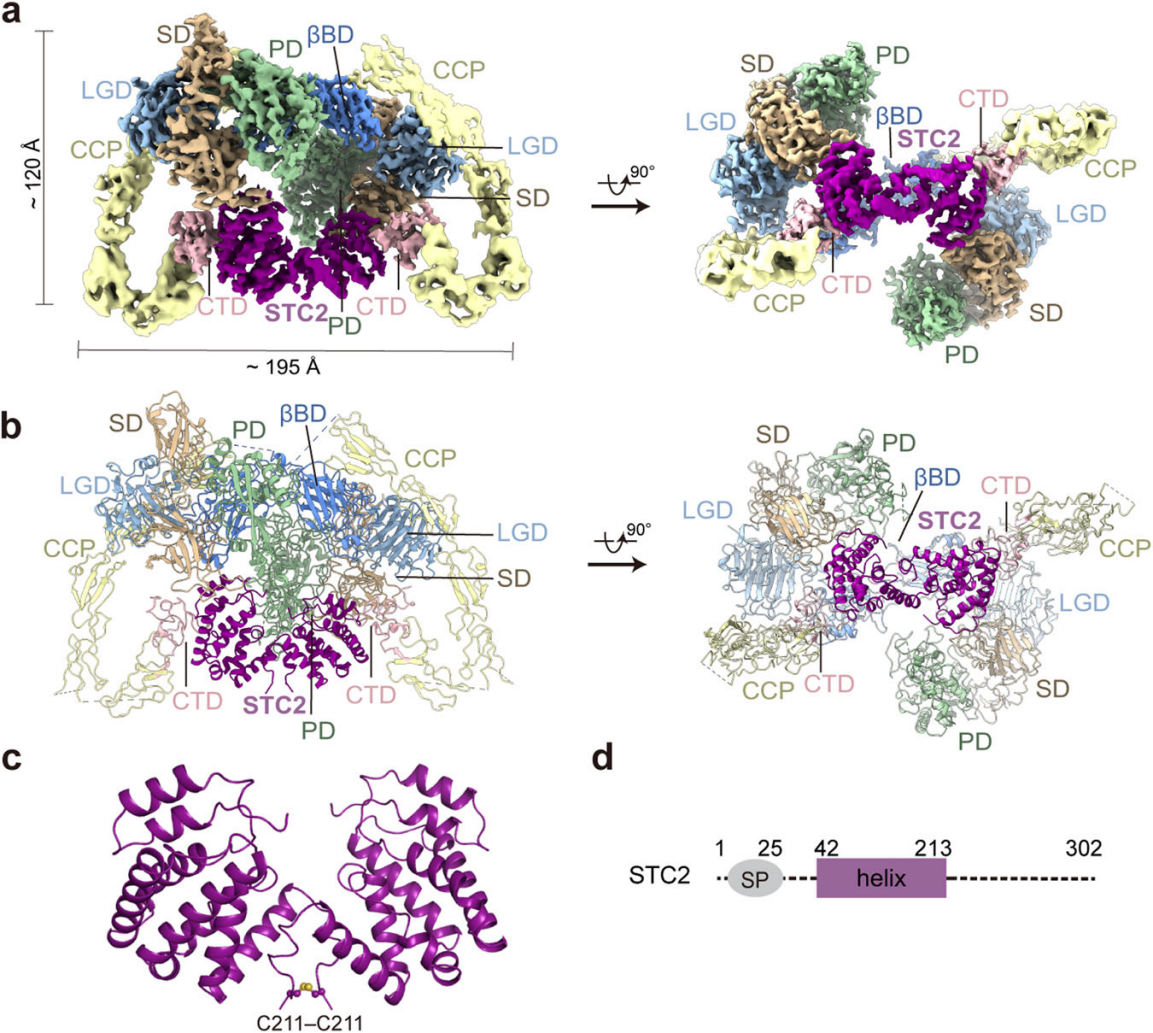
The cryo-EM structure of the PAPP-A·STC2 complex. **a** The overall color-coded EM density map of the PAPP-A·STC2 complex. **b** The corresponding cartoon representation. **c** The structure of the covalently linked STC2 dimer. **d** Schematic domain organization for STC2.

Next, we recombinantly overexpressed PAPP-A in suspended HEK293F cells to biochemically study the enzymatic properties of PAPP-A. The conserved core catalytic Glu483 residue was mutated to Alanine (E483A) to avoid auto-cleavage and serve as the loss-of-function control^34^. After the proteins were purified to homogeneity (Supplementary Fig. S5a–c), cryo-EM was applied and a final 3.8 Å-resolution half-density map was obtained using a similar strategy as for PAPP-A·proMBP (Supplementary Fig. S5d–i). The map revealed an extra component in the structure (Supplementary Fig. S6a), which was then identified to be the co-purified endogenous STC2 (Fig. 3a; Supplementary Video S2). Unlike the two proMBP subunits that locate separately in the PAPP-A·proMBP complex, STC2 exits as a homodimer^27^ and also forms 2:2 heterotetramer with PAPP-A (Fig. 3b; Supplementary Fig. S6b). Each STC2 contains nine α-helices and 14 cysteines, 12 of which form six pairs of intramolecular disulfide bonds and Cys211 residues from the two STC2 molecules form an intermolecular one to facilitate the dimerization (Fig. 3c). Besides, Cys120 links to Cys652 of PAPP-A and locates at the PAPP-A–STC2 interface (Supplementary Fig. S6c). All the inter- and intra-molecular disulfide-bond positions in the two complexes were thoroughly analyzed based on the cryo-EM density and were also validated using mass spectrometry (Table 1; Supplementary Figs. S3h, i, S6c and Tables S3, S4).

**Table 1:** The cysteine residues and the disulfide bonds in PAPP-A complexes.

Protein	Domain	Cysteine pairing			
PAPP-A	LGD				
	PD (LNR1**–**2)	C247**–**C507	C252**–**C577	C334**–**C348	C344**–**C360
		C377**–**C393	*C381***–***C51PM*	**C394–C405**	**C503–C542**
		**C520-unpaired**	C532**–**C563		
	SD	C630**–**C801	C633**–**C798	C652**–**C169PM/C120ST	
		C673**–**C755	C695**–**C701		
	βBD	C867**–**C895	C880**–**C891	C903**–**C910	C919**–**C931
		C956**–**C990	C971**–**C1059	C1112**–**C1125	*C1130***–***C1130*
	CCP1–5	C1135**–**C1189	C1147**–**C1158	C1162**–**C1200	C1205**–**C1249
		C1220**–**C1230	C1234**–**C1262	C1266**–**C1319	C1282**–**C1293
		C1297**–**C1330	C1335**–**C1378	C1348**–**C1358	*C1362***–***C1391*
		*C1398***–***C1459*	C1412**–**C1422	C1426**–**C1474	
	CTD (LNR3)	C1478**–**C1496	C1487**–**C1503	C1504**–**C1528	C1520**–**C1526
proMBP	–	*C51***–***C381PA*	**C89–C128**	C104**–**C107	C125**–**C220
		**C147-unpaired**	C169**–**C652PA	C197**–**C212	**C201-unpaired**
STC2	–	C56**–**C70	C65**–**C85	C76**–**C125	C109**–**C139
		C120**–**C652PA	C146**–**C181	C197**–**C205	C211**–**C211

Bold: firstly identified in our structure; underlined: intermolecular; italic: weak density or invisible.

*PM* proMBP, *ST* STC2, *PA* PAPP-A.

### PAPP-A alone is a highly flexible dimer

To eliminate the endogenous STC2 contamination and purify apo PAPP-A proteins, we constructed an STC2-knockout (KO) HEK293T cell line utilizing the standard CRISPR-Cas9 technique (Supplementary Fig. S7a–c). Although apo PAPP-A has been purified to a high homogeneity (Supplementary Fig. S7d–f), structural analysis failed to generate a high-resolution map due to the large structural flexibility. 2D classification showed that apo PAPP-A is still a dimer but the two subunits are flexibly attached together (Supplementary Fig. S7g). Similarly, during the data sorting of the two cryo-EM datasets, a population of particles suspiciously resembling monomer are actually PAPP-A dimer that presents pronounced instability between the two halves (Supplementary Figs. S2, S5). This observation indicates that both proMBP and STC2 play a role in stabilizing the PAPP-A dimer to adopt a relatively rigid conformation.

### ProMBP and STC2 bind to PAPP-A using similar mechanisms

The overall binding properties of proMBP and STC2 are similar as they are both embedded in PAPP-A and PAPP-A uses a similar set of domains to interact with them (half of the dimer were shown for simplicity) (Supplementary Fig. S8a, b). In the PAPP-A·proMBP complex, the binding of proMBP induces a nearly 30° rotation of the βBD towards the PD (Supplementary Fig. S8c), generating an interface between the βBD and the C-terminal subdomain of PD (Supplementary Fig. S7d). In contrast, the PD from PAPP-A·STC2 complex is only attached to the SD without interacting with any other domains. As a result, the relative orientation of the LGD, PD, and SD remains unchanged in the two complexes, forming a rigid core of PAPP-A; but the βBD, CCP, and CTD have drastic translational and rotational differences and thus constitute a movable region (Supplementary Fig. S8e). The comparison of the two structures identified a twist motion for PAPP-A conformation similar to a “butterfly wing-flap” (Supplementary Video S3).

**Fig. 4:**
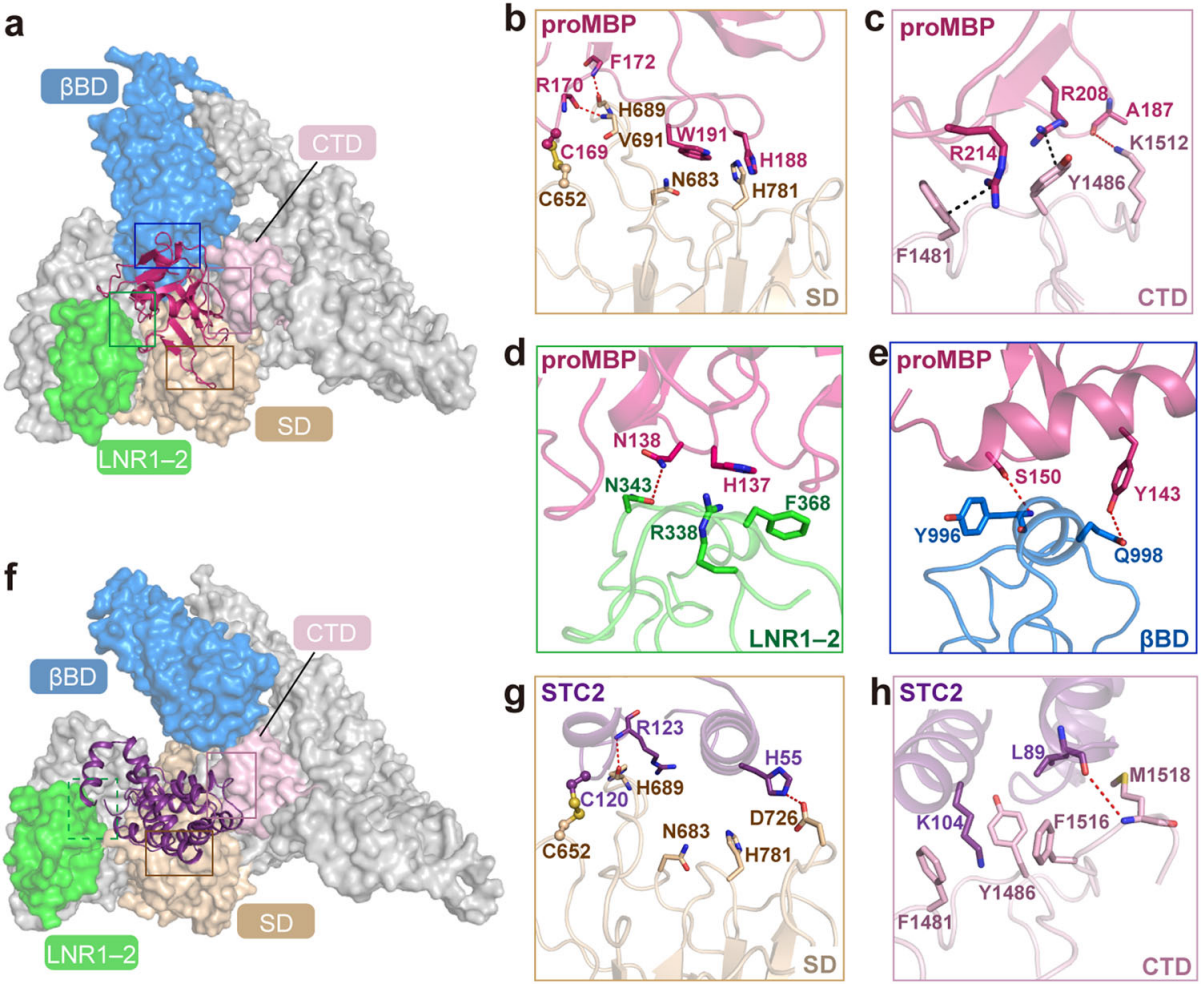
Structural properties of the binding interfaces between PAPP-A and proMBP or STC2. **a** The representation of the four interfaces between PAPP-A and proMBP with PAPP-A shown in color-coded surface and proMBP in the cartoon. The boxes represent the close-up details shown in **b**–**e**. **b** The interface between proMBP and SD. Cys169 of proMBP and Cys652 of SD that form a disulfide bond are shown as ball-and-stick. The indole of Trp191 insets into a hydrophobic groove of the SD. Arg170 and Phe172 of proMBP form two hydrogen bonds with Val691 and His689 of SD, respectively (red dashed lines). **c** The interface between proMBP and CTD. Arg214–Phe1481 and Arg208–Tyr1486 form two cationic–π stacks (black dashed lines) and Ala187–Lys1512 form a hydrogen bond. **d** The interface between proMBP and LNR1–2. Side chains of His137 and Asn138 from proMBP and Arg338, Asn343, and Phe368 from LNR1–2 form several hydrogen bonds and hydrophobic stacks. **e** The interface between proMBP and βBD. Two perpendicular helices form several pairs of interactions that include Tyr143 of proMBP, Gln998 of βBD, Ser150 of proMBP, and Tyr996 of βBD. **f** Structural representation of the interactions between PAPP-A and STC2 (same view as in **a**). The close-up views are presented in **g** and **h**. **g** The interface between STC2 and SD (same view as in **b**). Cys120 of STC2 and Cys652 of PAPP-A form a disulfide bond. Two other hydrogen bonds are formed by Arg123 and His55 from STC2 and His689 and Asp726 from SD, respectively. **h** The interface between STC2 and CTD (same view as in **c**). The side chain of Lys104 from STC2 inserts into a groove formed by Phe1481, Tyr1486, and Phe1516, creating a strong interaction. The main chain of Met1518 of PAPP-A and Leu89 of STC2 form a hydrogen bond.

In detail, proMBP is surrounded by four parts of PAPP-A: the SD, LNR1–2 from the PD, βBD and the CTD (Fig. 4a). In addition to the previously reported covalent linkage between PAPP-A-Cys652 and proMBP-Cys169^32^, we also found that several other residues of PAPP-A in this region, such as Asn683 and His689, contribute to the stabilization of this interface (Fig. 4b). Besides, we identified three new interfaces that have not been reported yet: (i) the CTD–proMBP interface which is mainly composed of two pairs of cation–π interactions (Arg214–Phe1481 and Arg208–Tyr1486) (Fig. 4c); (ii) the interaction of proMBP with LNR1–2 which is dominated by polar interactions involving a few highly charged acidic and basic residues from the two proteins (Fig. 4d); (iii) the stacking of proMBP on βBD which is formed by two perpendicular helices from the two proteins (Fig. 4e). However, as the pre-sequence (residues 1–87) of proMBP is invisible, we did not observe another previously reported disulfide bond between Cys51 of proMBP and Cys381 of PAPP-A^32^, yet its existence is confirmed by our disulfide-bond mass spectrometry (Table 1).

The rotation of βBD results in only three intermolecular interfaces between PAPP-A and STC2 that involve the SD, CTD, and LNR1–2 (Fig. 4f). The interface of SD with STC2 is similar to that with proMBP where Cys120 of STC2 is linked to the same Cys652 of PAPP-A (Fig. 4g), and the interface of STC2 with CTD comprises a hydrogen bond involving Met1518 and π–cation interactions involving Phe1481, which also resembles the CTD–proMBP interface (Fig. 4h). Unlike PAPP-A·proMBP complex, the LNR1–2 density in PAPP-A·STC2 complex is relatively less resolved (Supplementary Fig. S8f), indicating that its interaction with STC2 is probably weaker.

### ProMBP or STC2 inhibits the substrate cleavage by steric hindrance

**Fig. 5:**
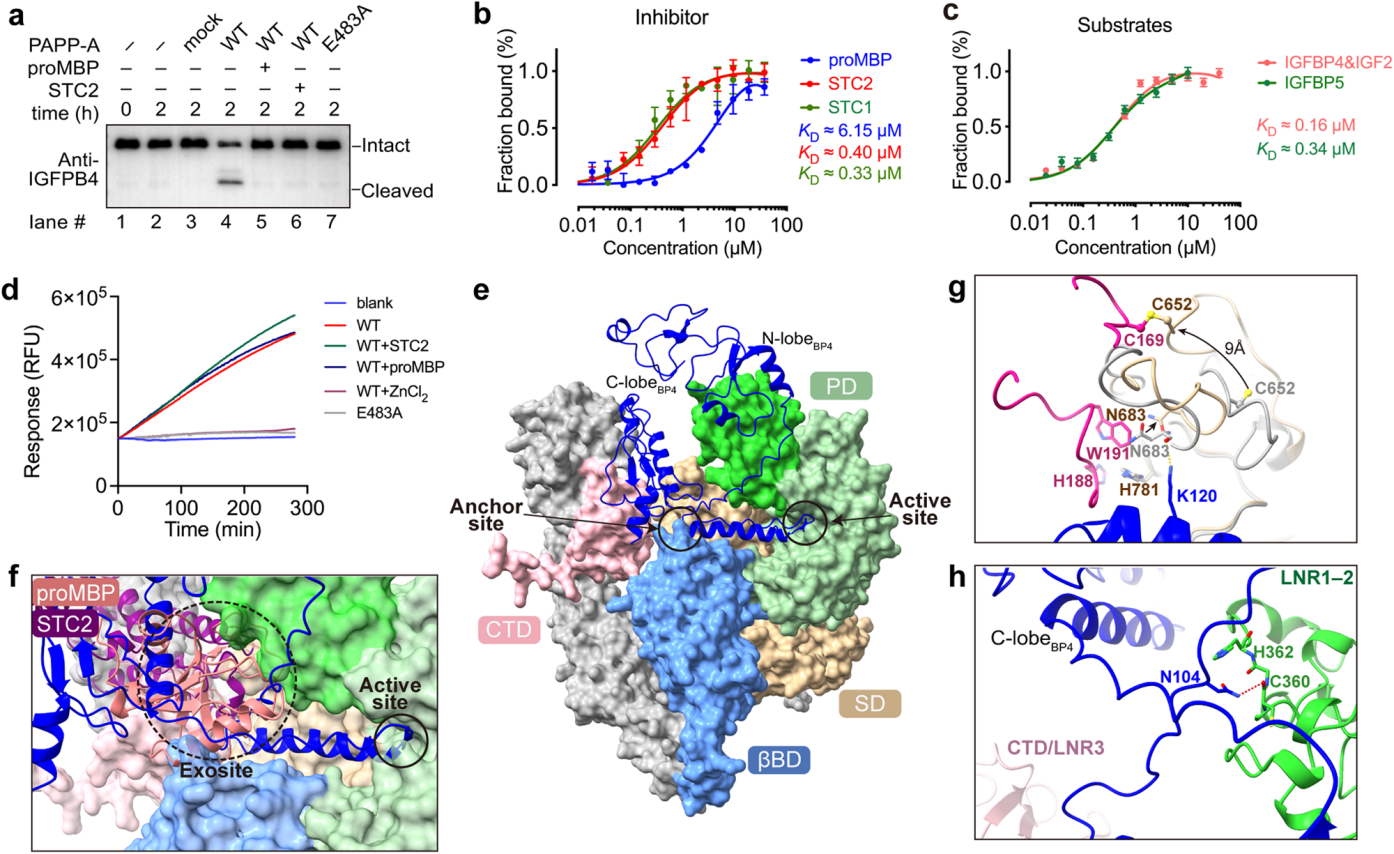
Proteolytic inhibition of PAPP-A activity requires the exosite binding of proMBP or STC2. **a** Cleavage of the full-length IGFBP4 in the presence of IGF-2 was assessed using in vitro reaction. The bands of intact and cleaved IGFBP4 detected by western blot analysis are indicated. Reactions with empty vector (mock) and inactive PAPP-A (E483A) were used as negative controls. **b** Microscale thermophoresis (MST) analyses of proMBP, STC1, and STC2 bound to PAPP-A. The identical batch of PAPP-A protein was used. The signals were consonant with the bound fraction, generating a dissociation constant (*K*_D_) that was measured from three biologically independent repeats (*n* = 3). **c** MST analyses of substrates bound to PAPP-A. *K*_D_ was measured from three biologically independent repeats (*n* = 3). **d** Fluorescence resonance energy transfer (FRET) analyses of PAPP-A cleavage on 4P1 under different inhibitory conditions. The absence of PAPP-A (blank), inactive PAPP-A (E483A), and the addition of ZnCl_2_ (WT + ZnCl_2_) were used as negative controls. **e** AlphaFold-predicted IGFBP4·PAPP-A complex model with IGFBP4 shown in cartoon (blue) and PAPP-A shown in surface (color-coded). **f** ProMBP or STC2 competes with the substrate for binding PAPP-A at the exosite. **g** Close-up view of proMBP and IGFBP4 binding on PAPP-A. SD is shown either in tan (in the PAPP-A·proMBP complex) or gray (in the predicted model). **h** In the predicted IGFBP4·PAPP-A structure, His362 of LNR1–2 inserts into a groove formed by a linker between the N-lobe and the anchor peptide, and Asn104 of IGFBP4 forms a hydrogen bond with the main chain of Cys360 of LNR1–2.

We next assessed the inhibitory properties of proMBP and STC2 towards PAPP-A. The STC2-KO cell line was used for preparing the PAPP-A variants (Supplementary Fig. S9a). As the full-length wild-type (WT) PAPP-A would efficiently cleave the substrate IGFBP4, the addition of either proMBP or STC2 strongly inhibited the cleavage reactions, to the extent of the inactive PAPP-A (E483A) (Fig. 5a). The binding properties of the modulators towards PAPP-A were then evaluated by microscale thermophoresis (MST), which demonstrated that the in vitro binding ability of STC2 was similar to that of STC1 (STC2 paralogue that also inhibits PAPP-A activity without formation of disulfide bonds^5^), with a dissociation constant (*K*_D_) of ~0.3–0.4 μM, over ten-fold higher than that of proMBP (*K*_D_ of ~6.15 μM) (Fig. 5b), suggesting a more robust binding of STC2 than proMBP. As the binding sites of proMBP or STC2 are nearly 40 Å away from the active-site cleft (Supplementary Fig. S9b), we hypothesize that these non-enzymatic domains of PAPP-A which the modulators interact with may also be involved in the binding of natural substrates. Each IGFBP substrate is composed of an N-lobe and a C-lobe connected by a flexible linker that contains the cleavage site (Supplementary Fig. S9c). PAPP-A-mediated cleavage of IGFBP4 is dependent on the presence of IGF, which is not the case for IGFBP5^35^. We found that both substrates bind to PAPP-A with similar affinities (Fig. 5c). Since IGFBP4 and IGFBP5 possess different linkers, we constructed several peptides derived from their linker regions containing the cleavage site (4P1–4P3 for IGFBP4 and 5P1–5P3 for IGFBP5; Supplementary Fig. S9d), which were inspired from the references^36,37^. Interestingly, their binding affinities were ~10–1000-fold lower than those of the full-length substrates; and with the gradual shortening in length and reduction of previously reported basic residues from 4P1 to 4P3 (or 5P1 to 5P3), their binding affinities to PAPP-A also decreased accordingly (Supplementary Fig. S9e, f). These results suggest that the binding of IGFBPs to PAPP-A is primarily mediated by the N- and C-lobes and that the interactions via the linker region are relatively weak.

To examine whether these peptides could be efficiently processed, we employed the fluorescence resonance energy transfer (FRET) technique and found that PAPP-A’s proteolytic activity was most potent towards 4P1, followed by 5P1 and 4P2, and the other peptides were barely cleaved (Supplementary Fig. S9g). This indicates that PAPP-A is incompetent in processing peptides with significantly shortened lengths. Therefore, 4P1 was chosen as a peptide substrate model for further analysis. As negative controls, both inactive PAPP-A variant and WT PAPP-A with ZnCl_2_ treatment had no cleavage activity (Fig. 5d). Interestingly, proMBP or STC2 conferred nearly no inhibition on the activity of WT PAPP-A towards 4P1 (Fig. 5d), which was sharply different from the cleavage of the intact substrate (Fig. 5a).

During the revision of our manuscript, Judge et al.^38^ reported the cryo-EM structure of IGFBP5-bound PAPP-A complex (PAPP-A_BP5_) and discovered that a so-called anchor peptide (Pro119 to Ser143) of the substrate binds at a substrate-binding groove. To test whether this is also the case for IGFBP4, we predicted the structure of the IGFBP4-bound PAPP-A complex using AlphaFold-multimer (v2.2.0) with the smaller genetic database (reduced_dbs) selected^39^. Indeed, a similar anchor peptide (Arg115 to Met135) is predicted to be embedded in the same groove in which one end is mediated by βBD/SD and the other end by PD, and the N- or C-lobes of IGFBP4 are dangling outside (Fig. 5e). Overlay of this predicted IGFBP4·PAPP-A complex model with our cryo-EM structures demonstrated that the modulators sterically compete with the substrate for binding to PAPP-A outside the catalytic domain (namely exosites^40^) (Fig. 5f). The K128_BP5_/K120_BP4_ anchor residues interact with Asn683 and His781 of PAPP-A, both of which are exactly located at the interface between proMBP (or STC2) and PAPP-A (Figs. 4c, h, and 5g). Thus, upon the formation of the disulfide bond between Cys169 of proMBP and Cys652 of PAPP-A (or Cys120 of STC2 and Cys652 of PAPP-A), it is expected that the binding of the anchor peptide would be prohibited (Fig. 5g). In addition, the predicted model showed that LNR1–2 region interacts with IGFBP4 via several hydrogen bonds and CTD/LNR3 is too far away to form any interaction (Fig. 5h), but the relatively less defined densities of LNR1–2, LNR3, and the N/C-lobe of IGFBP5 prevented us from examining these predicted interactions in our density map. Nonetheless, we could conclude that the non-enzymatic domains of PAPP-A provide exosites to facilitate the binding of IGFBPs, and once proMBP or STC2 bind to PAPP-A via multiple interfaces, the induced conformational change, and the direct steric competition would lead to the inhibition of PAPP-A cleavage.

### Substrate inhibition by proMBP or STC2 is independent of the oligomerization state of PAPP-A

Although the highly intrinsic flexibility at the interface between the two PAPP-A subunits results in a less resolvable density here, we still found that the dimeric interfaces contain not only the previously characterized intermolecular disulfide bond but also a strong hydrophobic pocket between the two βBD (Supplementary Fig. S10a). The monomeric variant (C1130S) was able to completely eliminate the formation of homodimer (Supplementary Fig. S10b, c, lanes 2 vs lanes 3), consistent with a prior result^32^. We then evaluated the complex formation and the inhibitory property of the modulators towards these PAPP-A variants. As shown in our structure (Fig. 1b), proMBP itself is a monomer. While proMBP formed a 2:2 tetramer with WT PAPP-A (Supplementary Fig. S10b, lane 4), it could only partially form a 1:1 dimer with C1130S variant (Supplementary Fig. S10b, lane 5). In contrast, the dimeric STC2 efficiently induced the formation of a 2:2 heterotetramer with the C1130S variant of PAPP-A (Supplementary Fig. S10c, lane 5).

The proteolytic assay revealed that C1130S still retained a comparable activity towards IGFBP4/IGF-2 as the WT PAPP-A (Supplementary Fig. S10d, top panel, lane 4–5), similar to a previous observation^30^, suggesting that the covalent dimerization of PAPP-A is not necessary for the regulation of PAPP-A activity towards IGFBP4. Moreover, in the presence of either proMBP or STC2, the protease activity of the monomeric mutant is similarly inhibited as WT PAPP-A (Supplementary Fig. S10d, lane 4 vs lane 5), demonstrating that the modulator-mediated inhibition is not dependent on the dimer formation of PAPP-A. Taken together, our studies disclose that the modulators inhibit PAPP-A through direct competition with the substrate for the non-catalytic exosites independent of the dimerization of PAPP-A.

### Aberrant regulation of PAPP-A activity by proMBP in EVT lineage is closely related to abnormal placentation

**Fig. 6:**
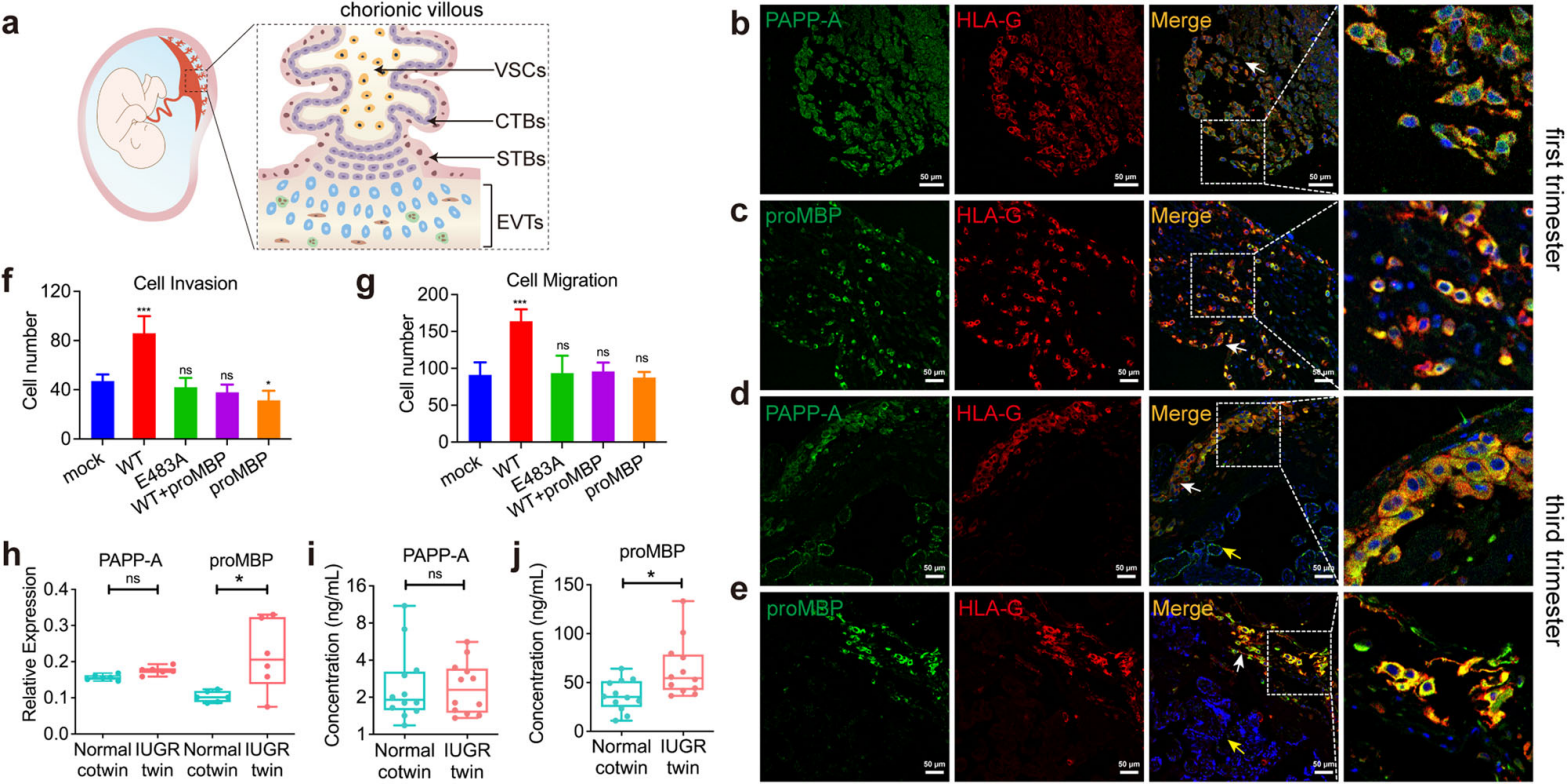
The modulation of PAPP-A by proMBP regulates the placental EVT function in sIUGR. **a** Schematic illustration of a placental villous anchored to the maternal decidua during gestation. Atop the villous stroma cells (VSCs), the cytotrophoblasts (CTBs) undergo cell fusion to produce the outer multi-nuclear syncytiotrophoblasts (STBs), which then directly contact maternal blood. EVTs are located outside the villi and invade the maternal decidua. **b**–**e** Co-staining IF analysis of PAPP-A, proMBP, and HLA-G in first-trimester decidual tissue section (8th week) (**b**, **c**) and term placental tissue section (40th week) (**d**, **e**). HLA-G human leukocyte antigen G. White arrows, decidual face; yellow arrows, chorionic face. The inset dashed squares indicate a higher-magnification view. Scale bars, 50 μm. **f**, **g** Cellular invasion (**f**) and migration (**g**) assays of HTR8/SVneo cells under different conditions. Compared to the mock, *P < 0.05; ***P < 0.005; ns, non-significant. **h** The relative protein expressions of PAPP-A and proMBP in placentas from the IUGR-twin and Normal-cotwin in sIUGR. **i**, **j** Protein concentrations of PAPP-A (**i**) and proMBP (**j**) in umbilical cord blood from the IUGR-twin and Normal-cotwin in sIUGR.

As PAPP-A and proMBP are abundantly expressed in the placenta, we then evaluated the function of PAPP-A proteolytic activity and the modulation of proMBP on placentation. The multilayered placenta is the only maternal-fetal interface that is composed of several specialized trophoblast cell types (Fig. 6a), while placental dysfunction results in various types of pregnancy complications and fetal anomalies^41^. We first examined the distribution of PAPP-A and proMBP using immunofluorescence (IF). PAPP-A is markedly co-localized with human leukocyte antigen G (HLA-G), a specific biomarker of EVTs in the placenta, from both first- and third-trimester pregnancies (Fig. 6b, d). A similar co-localization was observed for proMBP and HLA-G (Fig. 6c, e), indicating a possible regulatory function of PAPP-A and proMBP in EVTs. To gain insight into the effect of PAPP-A and proMBP on EVTs, we performed the transwell assay and found that the overexpression of WT PAPP-A promoted both migration and invasion of the HTR8/SVneo cells without influencing its proliferation, but this effect was abrogated by the presence of proMBP, similar to that of inactive PAPP-A (Fig. 6f, g; Supplementary Fig. S11a–c). As the inhibitory complex formation was detected in the culture medium (Supplementary Fig. S11d), the abrogated promotion of proMBP may be caused by its covalent inhibition towards PAPP-A. The slightly compromised cell invasion by the presence of proMBP alone was probably caused by its inhibition of endogenous PAPP-A activity (Fig. 6f).

Since the sIUGR twins possess identical genetic backgrounds and maternal environments, sIUGR is usually characterized by attenuated trophoblast invasion^42^ and is widely used for the investigation of trophoblast function. Our high-throughput proteomic analysis of placentas from sIUGR exhibited little difference in the expression levels of PAPP-A between the IUGR-twin and its normal cotwin (Normal-cotwin), but proMBP was significantly elevated in the IUGR-twin (Fig. 6h; Supplementary Table S5). This phenomenon was also observed from the published single-cell transcriptomic data that showed extremely high transcriptional levels of *PAPP-A* and *PRG2* in EVTs (Supplementary Fig. S11e)^43^. We also found that *IGF-2* is much more abundant in the EVTs (Supplementary Fig. S11e), consistent with the finding that IGF-2 is required for the continuous expansion of the placental microvasculature and fetal growth^44^. The expression patterns of PAPP-A and proMBP in umbilical cord blood of sIUGR twins were similar to those in the placentas, which displayed unchanged PAPP-A levels but elevated proMBP concentration in the IUGR-twin (Fig. 6i, j; Supplementary Table S2). These results cooperatively suggest that the overexpression of proMBP in the IUGR-twin of sIUGR would suppress the PAPP-A activity and impair EVT implantation, therefore resulting in the imbalanced growth of sIUGR twins, highlighting the significance of proMBP regulation of PAPP-A proteolysis on placental function.

## Discussion

**Fig. 7:**
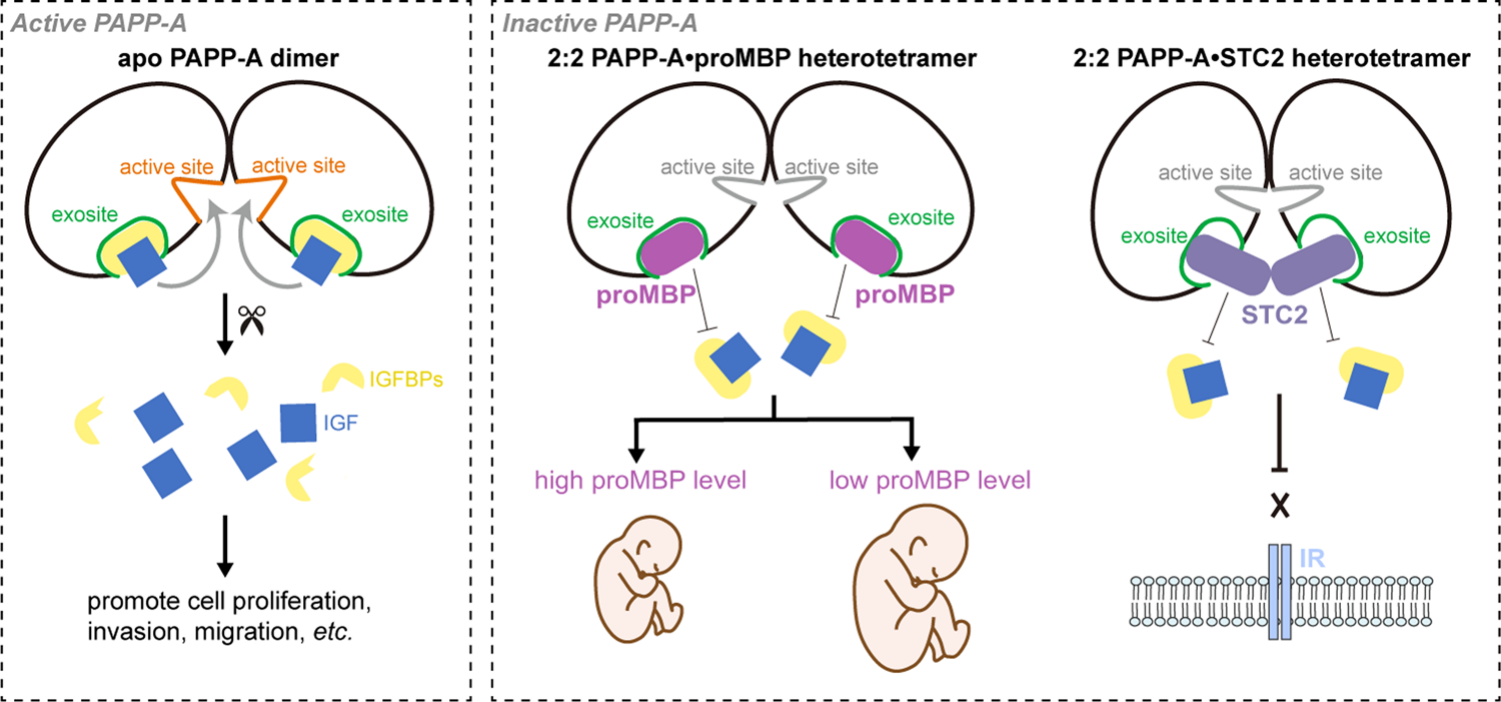
A simplified model for the regulation of PAPP-A activity by proMBP or STC2. Left, active PAPP-A dimer is able to bind the IGFBP**–**IGF at the exosites and cleaves IGFBP at the active sites, releasing the bioactive IGF to promote cell proliferation, cell invasion, cell migration, etc. Right, PAPP-A is inactive when complexed with either proMBP or STC2 at the exosites. Higher proMBP concentration leads to compromised PAPP-A activity and restricts fetal growth. STC2 could also modulate PAPP-A activity and block the downstream IGF receptor (IR)-mediated signaling.

As a key physiopathological molecule, PAPP-A and its downstream IGF signaling pathway members are promising diagnostic biomarkers and therapeutic targets in pregnancy and cancer^45,46,47^. The activity of PAPP-A is fine-tuned by proMBP or STC2 through covalent association, yet the structural and molecular basis remains largely elusive. In this study, the atomic cryo-EM structures of PAPP-A·proMBP and PAPP-A·STC2 complexes and the relevant biochemical and biological data reveal a wealth of information for the mechanistic interpretation of the PAPP-A’s proteolytic activity and functional involvement in placental disorders (Fig. 7).

In gestation, the autocrine function of PAPP-A expressed by EVTs is to promote the migration and invasion of the placenta. As proMBP is simultaneously expressed with PAPP-A, the complex assembly may either occur intracellularly or outside cells after secretion. Compared to the co-expression experiments described in Fig. 5a, the addition of the purified modulator proteins to PAPP-A-transfected cells completely failed to inhibit the activities of all variants (Supplementary Fig. S12a). This indicates that the extracellular environment, at least the medium, is not conducive to covalent complex formation. The superimposition of LG4 from laminin-α2 (A2LG4) on LGD shows that the glycan-binding site of A2LG4^48^ is co-localized with the interface between LGD and CCP2 (Supplementary Fig. S12b). If the LGD possesses oligosaccharide-binding ability as well, the CCP2–LGD interaction would mask its binding site toward cell surface glycosaminoglycan (GAG). As previously reported^49,50^, the highly basic cluster of negatively charged residues located at CCP2–4 may also mediate the cell surface adhesion through electrostatic interactions with GAG (Supplementary Fig. S12c). The mature form of proMBP is known to bind heparin; and in the PAPP-A·proMBP complex, this binding site is partially blocked by the CTD interaction (Supplementary Fig. S12d). Depending on these structural observations, we propose that both the active PAPP-A and proMBP would be tethered to the cell surface; when proMBP (or STC2) meets PAPP-A: (i) CCP2–LGD association may abolish the GAG-binding ability of LGD, (ii) CCP2–4 may adopt conformational changes to dissociate from GAG, and (iii) the CTD–proMBP interaction may release the heparin-binding of proMBP. Accordingly, the assembled PAPP-A·proMBP complex is detached from the cell surface and released into circulation, resulting in 99% of circulatory PAPP-A inhibited by proMBP and supporting the cell-dependent detachment of the complex^51^. PAPP-A is also reported to mediate the distal adipose tissue remodeling^52^, yet we do not completely understand how a covalently linked complex disassembles when it reaches adipose tissue and how PAPP-A rescues its proteolysis. Another possibility is that its function in adipose tissue is only mediated by the locally produced PAPP-A. If so, we still do not know why the placenta secretes such abundant PAPP-A and releases it into circulation. This paradox requires further investigation to uncover the disassembly rationale and the function of the inhibitory PAPP-A·proMBP complex in pregnancy.

Upon the traditional PAPP-A overexpression, we occasionally obtained the PAPP-A protein co-purified with the endogenous STC2 and solved the complex structure. On the contrary, STC1 was not detected in our system (Supplementary Fig. S13), indicating a more abundant expression of STC2 in HEK293F cells. However, the STC2-contaminated PAPP-A only accounted for a small amount, and the majority appeared as apo PAPP-A dimer that is too dynamic for the high-resolution structure determination (Supplementary Figs. S5, S7). Considering that the expression patterns vary significantly for proMBP and STC2 (Supplementary Fig. S14), we hypothesize that their binding may not be competitive and may bring out differential outcomes. Therefore, the proteolysis of PAPP-A must be fine-tuned to adjust its versatile roles under various conditions. Apart from modulating placental anomalies, some studies have delineated other physiological roles for PAPP-A, proMBP, and STC2. PAPP-A has a promoting function in cancer and longevity^7,53^. The degradation of IGFBPs by PAPP-A in preovulatory follicles might constitute a critical factor in leading to the selection of dominant follicles^54,55^. With the establishment of the tissue model and single-cell RNA profiles^56,57^, more precise roles for PAPP-A activity will be discovered. ProMBP is abnormal in large-for-gestational-age (LGA) and shows mislocation in fetal membranes^58,59^. STC2 is regarded to be highly related to human height, as some STC2 variants containing the height-increasing alleles display compromised proteolytic inhibition of PAPP-A, leading to increased cleavage of IGFBP4^60,61^. These novel findings illustrate that regulation of PAPP-A activity constitutes an extraordinarily important signaling pathway, and in turn, highlight the significance of our present structural interpretations and biochemical analyses about PAPP-A complexes.

In summary, our determination of atomic structures of PAPP-A·proMBP and PAPP-A·STC2 complexes revealed the molecular basis for PAPP-A’s proteolytic activity and the inhibitory mechanism underlying the covalent modulators. The correlation between PAPP-A–proMBP dysfunction and impaired EVT invasion in sIUGR provides the impetus for further studies on their cause-and-effect relationships. Combined with the key evolutionary role of the IGF system, our work provides a crucial framework for a proMBP(STC2) → PAPP-A → IGFBPs signaling cascade and suggests the possibility for future drug development and subsequent therapeutic applications.

## Materials and methods

### Protein purification of PAPP-A·proMBP complex from the pregnant plasma

We applied the endogenous purification as previously described^19^ with modifications. Approximately 20 mL plasma derived from ~50 pregnant women were added with 20 mM Tris, pH 8.0, followed by the addition of PEG6000 (Merck, 807491) to a final concentration of 10%. A protease inhibitor cocktail containing aprotinin at 1.3 μg/mL, pepstatin at 1 μg/mL, leupeptin at 5 μg/mL, and 1 mM phenylmethylsulfonyl fluoride (PMSF, Amresco, 0754) was added. After stirring at 4 °C for 1 h, the precipitate was removed by centrifugation at 4000 rpm at 4 °C for 20 min and further centrifugation at 12,000 rpm for another 20 min. The supernatant was filtered with a 0.45-μm filter membrane, loaded to ion exchange (SOURCE-15Q, GE Healthcare), and eluted with 20 mM Tris–Na, pH 8.0, and an increasing concentration of NaCl from 50 mM to 1 M as liner gradient. In order to determine which fractions contained the PAPP-A·proMBP complex, we applied western blot analysis with the PAPP-A antibody (Santa Cruz, sc-365226). Fractions that contained target proteins were collected and concentrated to 2 mL, and then MonoS Buffer A (20 mM Citrate–Na, pH 4.5, 50 mM NaCl) of 30 mL was slowly added to reduce the salt concentration. The resulting solution was then applied to ion exchange (MonoS 5/50 GL, GE Healthcare) and eluted with an increasing concentration of NaCl from 50 mM to 1 M. Finally, the protein was concentrated and applied to gel filtration chromatography (Superose-6 10/30, GE Healthcare) in the phosphate buffer solution (PBS, pH 7.4). The peak fractions were concentrated and stored at –80 °C.

### Plasmid construction, transient expression, and recombinant protein purification

The full-length human *PAPP-A* cDNA was optimized and synthesized, and the mature form (residues 1**–**1547) was sub-cloned into the pCAG vector with an IgG kappa signal peptide and an N-terminal maltose-binding protein (MBP)-His_10_ tag. Point mutations were introduced using a standard two-step polymerase chain reaction (PCR) and homologous recombination. All constructs were confirmed precisely by sequencing.

Human embryonic kidney (HEK) 293F cells were cultured in SMM 293T-II medium (Sino Biological Inc.) at 130 rpm at 37 °C under 5% CO_2_ in a Multitron-Pro shaker (Infors). When the cell density reached 2.0 × 10^6^ cells/mL, 2 mg of the plasmid was transiently transfected with 4 mg polyethylenimine hydrochloride (Polysciences) for each liter cell. 72 h later, the cell pellets were harvested by centrifugation at 6000 rpm for 20 min. The supernatant was collected and filtered using 0.45-μm microporous membrane, and further concentrated by vivaflow^®^200 (Sartorious) equipped with peristaltic pump (LONGER).

For purification of the recombinant PAPP-A, the concentrated cell medium was loaded to the Ni Smart Beads 6FF (Smart Lifesciences) equilibrated with lysis buffer (20 mM HEPES-K, pH 7.4, 150 mM NaCl, 2 mM CaCl_2_) and incubated at 4 °C for 15 min. The beads were washed three times with lysis buffer supplemented with 5 mM imidazole, and the bounded protein was eluted with lysis buffer plus 300 mM imidazole after incubation at 4 °C. The eluent was concentrated to ~5 mL and loaded on a HiTrap Heparin HP ion exchange (Cytiva) and separated with NaCl gradient. Finally, the eluted protein was applied to size exclusion chromatography (Superose-6 Increase 10/300 GL, GE Healthcare) in the PBS buffer.

### Cryo-EM sample preparation and data acquisition

Holy-carbon gold grids (Quantifoil, R1.2/1.3) were treated with Solarus 950 plasma cleaner (Gatan) with a 4:1 ratio of O_2_/H_2_ for 60 s for glow-discharge before cryo-EM sample preparation. 4 μL aliquots of freshly prepared PAPP-A·proMBP or PAPP-A·STC2 (0.2 mg/mL) complex were applied on the grids, blotted with filter paper (Whatman No. 1) with force set to –2 for 0.5 s at 4 °C and 100% humidity, and plunge-frozen in the liquid ethane using a Vitrobot Mark IV (FEI).

The cryo-grids were screened on a 200 kV Talos Arctica microscope equipped with an FEI Ceta camera and a K2 Summit direct electron detector (Gatan). Data collection was carried out with Titan Krios electron microscope (FEI) operated at 300 kV.

Images were recorded with a K2 Summit direct electron detector (Gatan) in the super-resolution mode at a nominal magnification of 130,000× and a dose rate of 8 e^−^/s/pixel. Movies were recorded semi-automatically using the SerialEM software^62^. A GIF Quantum energy filter (Gatan), with a slit width of 20 eV was used at the end of the detector. The defocus range was set from –0.7 to –1.2 μm. The total exposure time was 8.32 s, and intermediate frames were recorded every 0.26 s. 32 frames per image were acquired. Statistics for data collection are summarized in Supplementary Table S1.

### Imaging processing

For the PAPP-A·proMBP complex, a total of 9228 movie stacks were recorded (calibrated pixel size 1.052 Å). Raw movie frames were aligned and averaged into motion-corrected summed images by MotionCor2^63^. The Gctf program (v1.06)^64^ was used to estimate the contrast transfer function (CTF) parameters of each motion-corrected image. All the following data processing was performed with Relion^65^. 926 particles were manually picked and subjected to 2D classification to generate templates for automatic particle picking. A total of 1,497,910 particles were then auto-picked from the 8646 images that were manually selected from the motion-corrected images. The picked particles were subjected to one round of 2D classification, and 889,811 particles were selected for subsequent 3D classification. The initial model was generated from the selected particles with high quality. Two classes from the 3D classification were selected for 3D refinement with C2 symmetry imposed, resulting in a map at an overall 3.64 Å resolution after mask-based post-processing (gold-standard FSC 0.143 criteria). To optimize the local density, symmetry expansion and focused classification/refinement were further performed and a final map was produced at 3.45 Å resolution. The local resolution map was analyzed using Relion and displayed using UCSF Chimera^66^. Workflow of the data processing was illustrated in Supplementary Fig. S2.

The processing of the PAPP-A·STC2 datasets (calibrated pixel size 1.055 Å) was similarly completed (Supplementary Fig. S5).

### Atomic model building and refinement

The PAPP-A model was manually de novo built using Coot^67^. Due to poorly resolved densities of the flexible regions, residues 1265–1396 of PAPP-A (mainly corresponding to CCP3–5) were rebuilt from a reference model generated by AlphaFold2^33^. The model of proMBP in the map of the PAPP-A·proMBP complex was homologously built based on a published structure (PDB code: 1H8U)^68^. The model of STC2 in the map of the PAPP-A·STC2 complex was de novo built depending on the sequences and the density map. The models for the PAPP-A·STC2 and PAPP-A·proMBP complexes were further refined in real space using PHENIX^69^. Model validation was summarized in Supplementary Table S1.

### Purification of IGFBP4

The cDNA for *IGFBP4* was sub-cloned into the pCAG with a C-terminal His_6_-tag. Similar to that of PAPP-A protein, the purification of the secreted IGFBP4 was achieved by Ni Smart Beads 6FF. After 15 min incubation of the supernatant, the beads were successively washed with lysis buffer plus 5 mM imidazole and eluted with lysis buffer plus 250 mM imidazole. The eluted protein was concentrated to ~2 mL with Amicon^®^ Ultra-15 ultrafiltration tube (Millipore) and applied to Superdex-200 10/300 GL (GE Healthcare) in PBS buffer.

### Construction of the STC2-KO HEK293T cells

The HEK293T cells were cultured in DMEM (C11995500BT, Gibco) containing 10% fetal bovine serum (FBS, 10099141C, Gibco). For transfection, cells were digested with 0.25% trypsin (Gibco) to single cells. The STC2-KO HEK293T cells were generated by traditional CRISPR-Cas9 gene editing. Two pairs of gRNAs (A1: TTGCAGCCCTTCACCGAATG(-TGG), A2: GGGTTTCGACCGGTCAGCTT(-GGG); and B1: CACCCGGATCCCTAATTAAA(-AGG), B2: GACCCTGGGATAACCGAAGT(-GGG)) were pre-incubated with Cas9 protein to form ribonucleoproteins and transfected into 293T cells by electroporation (Celetrix LLC). The monoclonal cell population was selected by limiting dilution. The genotype of knockout cells was confirmed by PCR, sequencing, and western blotting (Supplementary Fig. S7). Three pairs of primers were used for PCR (F1: CTCTGCTCTACCGGCTCTTG, R1: GGGCAATGCAGAGAGGTCAT; F2: CACTGTTTGGTCAACGCTGG, R2: CATCCTGTGGGATTGCCCTT; F3: CTCTGCTCTACCGGCTCTTG, R3: CATCCTGTGGGATTGCCCTT). Colony C8 was identified as homozygote.

### In vitro PAPP-A enzymatic cleavage of IGFBP4

We used the culture supernatant of various PAPP-A constructions and the recombinant IGFBP4 protein for enzymatic cleavage reaction. The protein expressions were standardized by western blot analysis using anti-MBP-tag antibody (EARTHOX, E022240-01) (Supplementary Fig. S9a). The empty vector was transfected and the corresponding medium was used as a control (mock). IGFBP4 was pre-incubated with access to IGF-2 (Sino Biological Inc.) on ice for 30 min. Then IGFBP4/IGF-2 proteins were mixed with an equal amount of various PAPP-A and incubated for 2 h at 37 °C. The total reaction volume was adjusted to 10 μL with PBS. The final concentrations in cleavage reactions were 800 nM for IGFBP4 and 4 μM for IGF-2. Reactions were then visualized by western blotting and probed for visualization with IGFBP4 antibodies (Abcam, ab205581). Background degradation was also assessed without the addition of any medium.

### Western blot analysis

Protein samples were resolved by SDS-PAGE and transferred to polyvinylidene fluoride (PVDF) membranes (IPVH00010, Millipore). The membranes were blocked by 5% (w/v) Skim Milk (232100, BD) at room temperature for 1 h and incubated with the primary antibodies at 4 °C overnight with an appropriate dilution ratio. Then the membranes were washed with the TBS-T buffer (25 mM Tris, pH 8.0, 150 mM NaCl, and 0.05% (w/v) Tween-20) for three times. The horseradish peroxidase (HRP) conjugated-secondary antibodies (P03S02M, Gene-Protein Link) were incubated for 1 h and washed similarly, and visualized under automatic chemiluminescence imaging analysis system (Tanon). All the uncropped gels were presented in Supplementary Fig. S15.

### MST

The equilibrium dissociation constant (*K*_D_) values for protein–protein and protein–peptide interaction were measured using the Monolith NT.115 Capillaries (NanoTemper Technologies, München, Germany). The purified PAPP-A was firstly adjusted to a concentration of 2 μM and then fluorescently labeled by Protein Labeling Kit RED-NHS (NanoTemper Technologies, Germany) according to the manufacturer’s protocol. Peptides, substrates, and modulator proteins were serially diluted in binding buffer (PBS buffer containing 0.05% Tween-20) and then mixed and incubated with an equal volume of 24 nM labeled PAPP-A at room temperature for 30 min. The sample was loaded into the NanoTemper glass capillaries and micro-thermophoresis was carried out using 20% light excitation power and medium MST power. The data were obtained from triplicate independent experiments and the *K*_D_ values were calculated using the signal from an MST-on time of 1.5 s by the NanoTemper Monolith affinity software using 1:1 binding mode. Flag peptide (DYKDDDDK) was used as a negative control without any signals (data not shown).

### FRET

We selected the linker regions of IGFBP4 and IGFBP5 as peptide substrates. The modified peptides were synthesized by Scilight-Peptide company (Beijing, China). In detail, modified peptides carry an *o*-aminobenzoic acid-modified lysine mutant residue [Lys(Abz)] at the position of Ser131 or Lys139, and a 3-nitrotyrosine-modified tyrosine mutant residue [Tyr(NO_2_)] at the position of Gly139 or Gly147 for IGFBP4 and IGFBP5, respectively. For the enzyme kinetics test, we monitored peptide cleavage using a fluorescence plate reader (EnVisoin, PerkinElmer, USA) in a 384-well plate (code 3570, Corning) by following the increase in relative fluorescence units (RFU) at 420 nm emission with 310 nm excitation, with buffer containing 50 mM Tris, pH 8.0 and 1 mM CaCl_2_.

### Mass spectrometry for the disulfide-bond formation

The PAPP-A protein samples (200 μg) were desugarized by PNGase F (P0704S, New England Biolabs) in ultracentrifuge tubes (PALL Corporation) at 37 °C overnight. After centrifugation at 11,000× *g* for 10 min, the free sulfhydryl group was blocked by *N*-ethylmaleimide (NEM, ES04260, JSENB). 10 μg Trypsin (V5117, Promega) was added and incubated at 37 °C for 4 h. Then, chymotrypsin (C4129, LABLEAD) was added and incubated at 37 °C overnight for an enzymatic hydrolysis reaction. After the enzymatic hydrolysis reaction, the sample was divided into two tubes. One tube was acidified by adding 1% FA for disulfide bond analysis. The other tube was added with 10 mM DTT (0281, AMRESCO) for reduction reaction, and was then alkylated with iodoacetamide (VT335, JSENB) at a final concentration of 20 mM followed by acidification with 1% FA.

For liquid chromatography with tandem mass spectrometry (LC–MS/MS) analysis, the peptides were separated by a 65-min gradient elution at a flow rate of 0.300 μL/min with the Thermo EASY-nLC1200 integrated nano-HPLC system which is directly interfaced with the Thermo Q Exactive HF-X mass spectrometer.

### Ethical approval, participants, plasma and placenta collections

The present study was reviewed and approved by the Institutional Review Board of Peking University Third Hospital (No. 2020-310-02) and the included puerperants were informed of our objectives and signed the appropriate informed consent. The remaining plasma after routine blood examination from women in late pregnancy (≥ 34 weeks) was collected and pooled. Placentas in the first trimester were obtained from pregnant women undergoing elective termination of pregnancy at 8 weeks of gestation, and those in the third trimester were obtained from women undergoing Cesarean section at term. The gestational age was determined by ultrasound. All the samples were obtained at the Department of Gynecology and Obstetrics, Peking University Third Hospital.

### IF analysis of paraffin-embedded tissues

Placental and decidual tissues were fixed with paraformaldehyde and embedded in paraffin. Serial sections were cut into 5 μm, deparaffinized and the antigens were retrieved. Subsequently, slides were blocked and incubated with primary antibodies overnight at 4 °C, and then washed and incubated with secondary antibodies. Images were acquired by a fluorescence microscope (LSM710, Zeiss). The antibodies used were: murine monoclonal antibodies against PAPP-A (Santa Cruz, sc-365226)^70^, rabbit monoclonal antibodies against PRG2 (Abcam, ab154655), and murine/rabbit monoclonal antibodies against HLA-G (Abcam, ab283260). Nuclei were stained with DAPI. Tissues were classified as decidua basalis when the prominent invasion of HLA-G^+^ EVTs was noticed. Three samples from each pregnancy group (first and third trimesters of pregnancy) were analyzed.

### Cell proliferation, migration, and invasion assays

Human trophoblast HTR8/SVneo cell line was kindly provided by Dr. Hongmei Wang from the Institute of Zoology, Chinese Academy of Sciences. HTR8/SVneo cells were cultured in 1640 medium supplemented with 10% characterized FBS, 10 units/mL penicillin and 10 mg/mL streptomycin (Millipore, USA) and maintained in humidified incubators at 37 °C with 5% CO_2_. Cells were transfected using Lipofectamine 3000 transfection reagent (Invitrogen, USA). Empty vector (mock) was transfected as a control.

Cell proliferation was measured by a Counting Kit-8 (CCK-8) detection kit (Cat # CK04, Dojindo Molecular Technologies, Japan). The cells were seeded in a 96-well plate and treated with plasmid transfection. At the indicated time points, 10 μL of CCK-8 solution was added, followed by incubation at 37 °C for 2 h, and the absorbance at 450 nm was determined.

After being transfected for 36 h, 150 μL resuspended cells (2 × 10^5^ per mL) were plated in the upper chamber of each transwell (Corning, New York), then inserted into the bottom chamber containing 800 μL complete 1640 medium (10% FBS) for migration assay. After 48 h culture, the transwells were fixed in methanol and stained with 0.1% crystal violet solution. Cells remaining in the upper chamber were removed. For invasion assay, transwells pre‐coated with matrigel (Corning, New York) were used instead of normal ones, while the other procedures were similar to those of migration assay. The polycarbonate membrane carrying penetrated cells was cut off, sealed with resin, and then observed under 200× microscope. Cells in captured images were counted with ImageJ and measured with SPSS 21.0 software.

### Enzyme-linked immunosorbent assay (ELISA)

Umbilical cord blood of the IUGR-twin and Normal-cotwin from sIUGR pregnancies were collected at delivery and frozen and stored at –80 °C. The thawed plasma was centrifuged at 13,000 rpm at 4 °C for 10 min. Supernatants were analyzed with the Human PAPP-A ELISA Kit (AL-101-I, AnshLabs) and Human PRG2 ELISA Kit (BJ12242020, Aviva Systems Biology) following the manufacturer’s procedures.

## Supplementary information

Supplementary Information

Supplementary Table S3

Supplementary Table S4

Supplementary Table S5

Supplementary Video S1

Supplementary Video S2

Supplementary Video S3

## Data Availability

The cryo-EM maps of 2:2 PAPP-A·proMBP heterotetramer, 1:1 PAPP-A·proMBP subcomplex (half map), 2:2 PAPP-A·STC2 heterotetramer, and 1:1 PAPP-A·STC2 subcomplex (half map) have been deposited at the EMDB with the accession codes of EMD-34738, EMD-33621, EMD-34739, and EMD-33622, respectively. The corresponding atomic models have been deposited at the RCSB PDB with the accession codes of 8HGG, 7Y5N, 8HGH, and 7Y5Q, respectively.
