# Supplementary material for: Structural insights into the covalent regulation of PAPP-A activity by proMBP and STC2
Source: Cell Discov. 2022 Dec 22;8:137. doi: 10.1038/s41421-022-00502-2 (PMC9780223; doi:10.1038/s41421-022-00502-2)
Supplement: Supplementary file 1 — Supplementary Information [file 41421_2022_502_MOESM1_ESM.pdf]

Supplementary Information

Structural insights into the covalent regulation of PAPP-A activity by proMBP and STC2

Qihang Zhong[1, 2, 3, 4], Honglei Chu[1, 2, 3, 4], Guopeng Wang[5], Cheng Zhang[6], Rong Li[1, 2, 3, 4], Fusheng Guo[7, 8], Xinlu Meng[1], Xiaoguang Lei[7, 8, 9], Youli Zhou[10], Ruobing Ren[10,11], Lin Tao[12], Ningning Li[5], Ning Gao[5, 8], Yuan Wei[1, 2, 3, 4] ✉, Jie Qiao[1, 2, 3, 4, 8] ✉, and Jing Hang[1, 2, 3, 4] ✉

Supplementary Fig. S1–S15 and legends

Supplementary Video S1–S3 titles

Supplementary Tables S1–S2

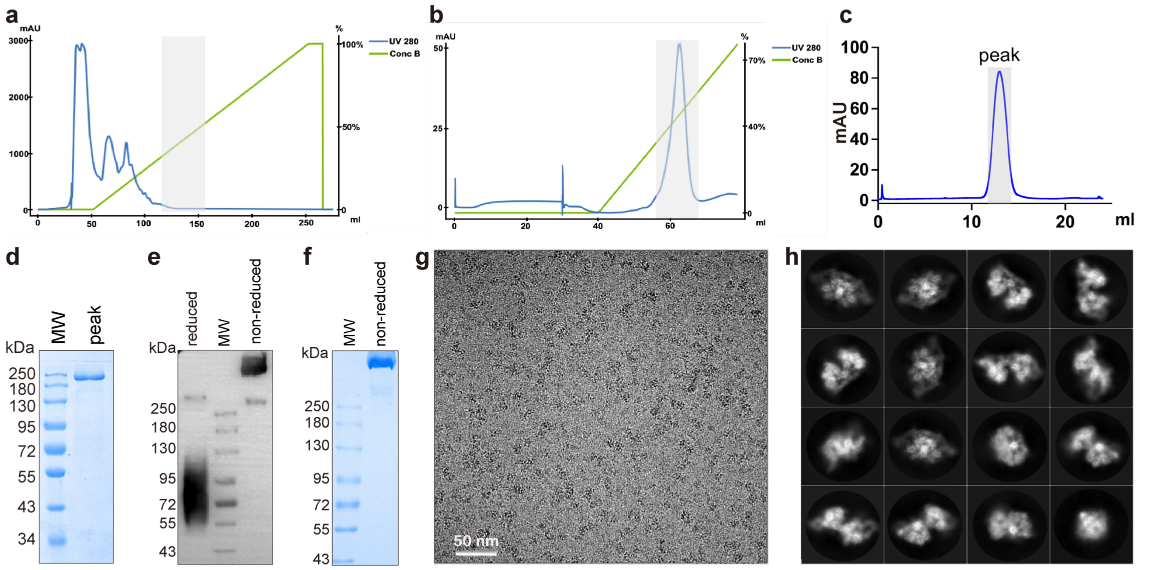

Supplementary Fig. S1 Endogenous protein purification and characterization of human PAPP-A•proMBP complex.

a–c, Representative purification of ion-exchange (a), cation-exchange (b), and size-exclusion (c) chromatography for PAPP-A•proMBP purification. **d,** Coomassie blue staining of the purified proteins. Details were presented in Methods. **e,** Purified proteins were separated on reduced (left) and non-reduced (right) SDS-PAGE and identified by western blot using antibody against proMBP. **f,** Coomassie blue staining of the purified proteins separated on non-reduced SDS-PAGE. **g–h,** A representative cryo-EM micrograph (g) and reference-free two-dimensional class averages (h) of the PAPP-A•proMBP complex from 300kV Titan Krios. Scale bar, 50 nm.

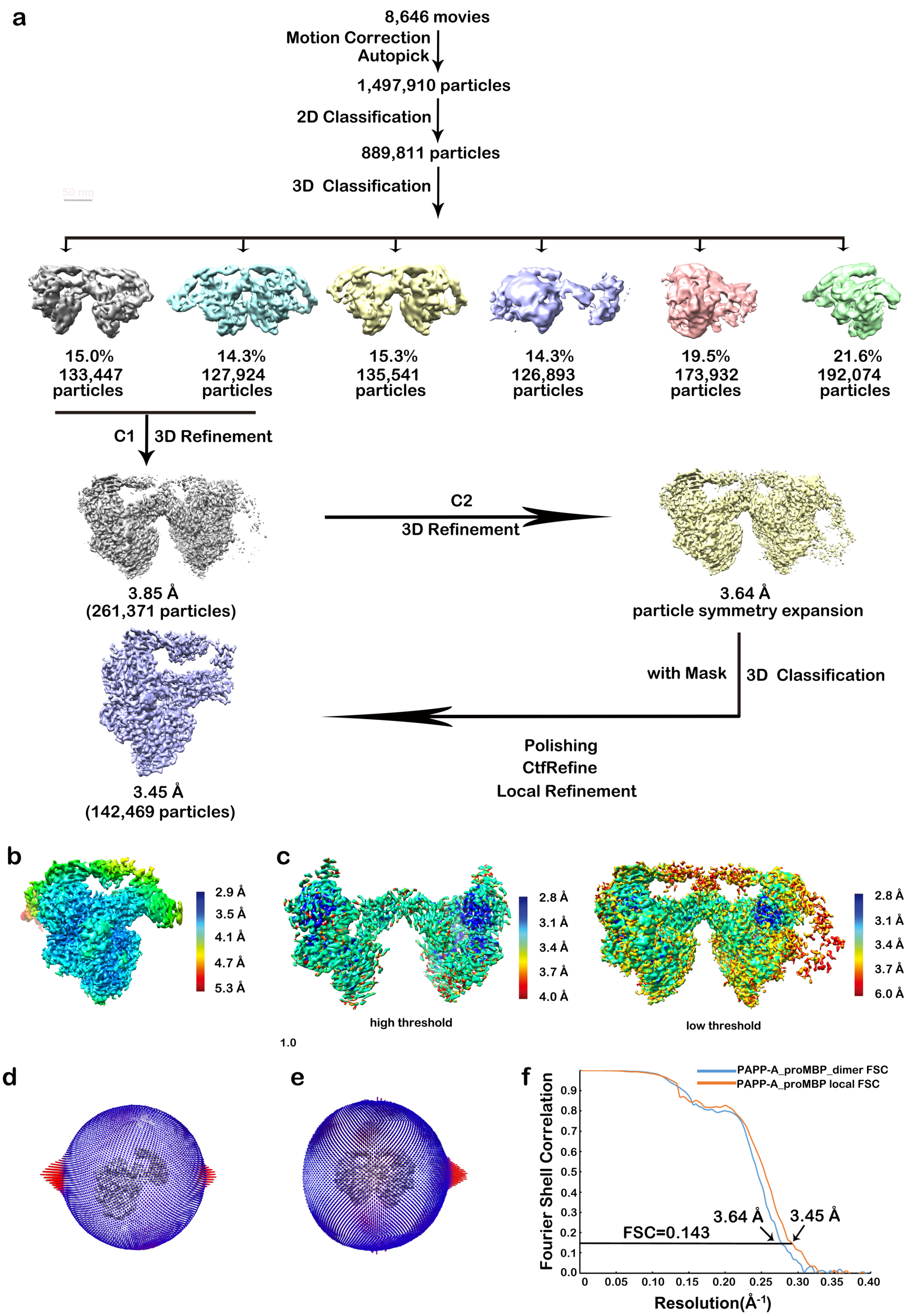

Supplementary Fig. S2 Cryo-EM data processing and structure determination of the human PAPP-A•proMBP complex.

a, Workflow of cryo-EM data processing of the PAPP-A•proMBP complex. **b–c,** 3D density maps for the half (b) and dimer (c) colored according to local resolution (Å). **d–e,** The orientation distributions for the particles from the dataset of the half (d) and dimer (e). **f,** Gold-standard Fourier shell correlation (FSC) curves with estimated resolution at 0.143 of the final density map of dimeric PAPP-A•proMBP complex (blue) and local half (orange).

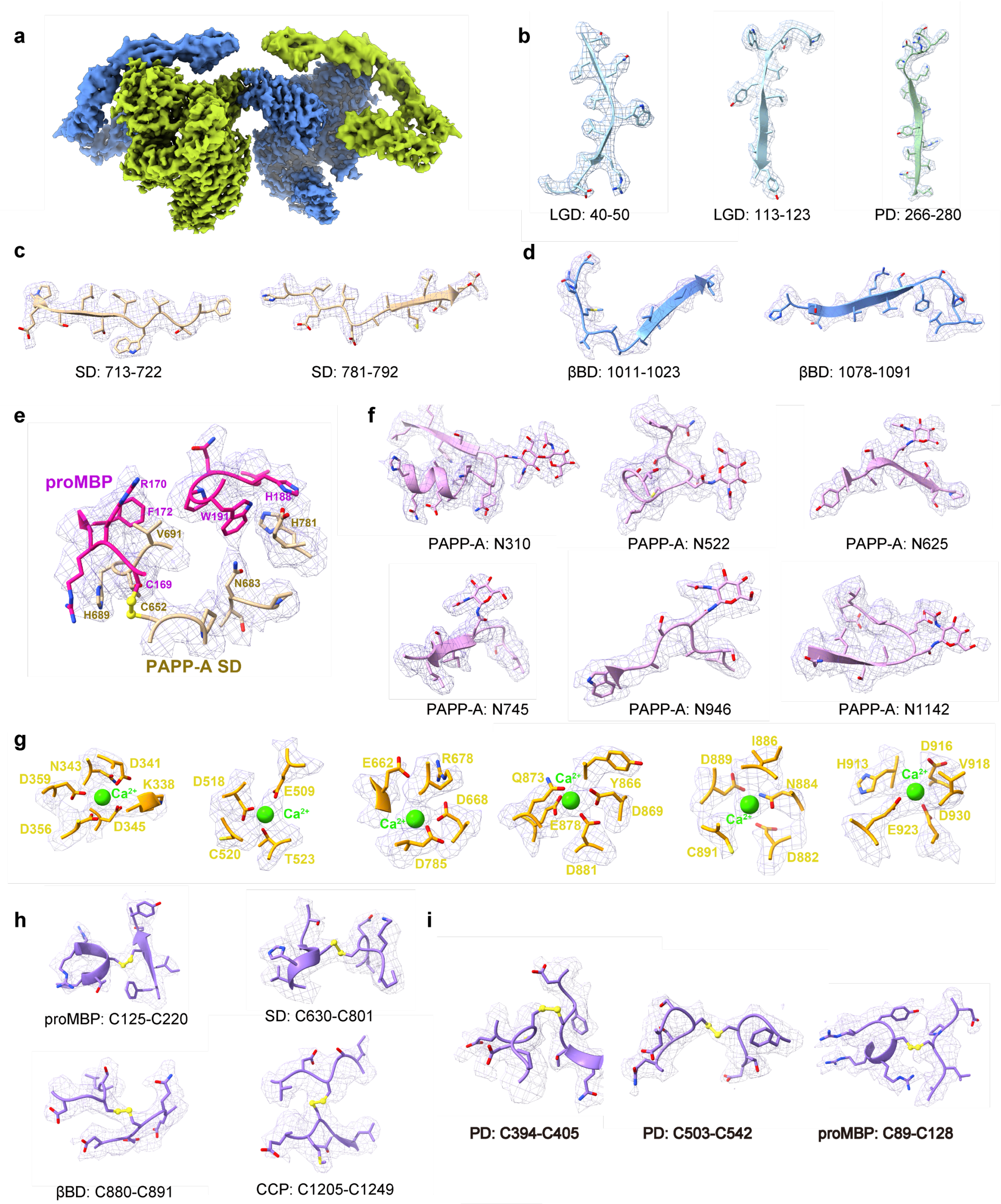

Supplementary Fig. S3 Local electron microscopy densities for the PAPP-A•proMBP complex.

a, The electron microscopy map for PAPP-A dimer with two PAPP-A subunits form *in trans*. **b,** Representative cryo-EM densities for PAPP-A. **c–d,** A close-up view for the representative EM densities of SD (c) and βBD (d) domains. **e,** The interface density between proMBP and SD. **f,** A close-up view for the representative cryo-EM densities of the glycosylation sites. **g,** A close-up view for the representative cryo-EM densities of ion coordination. **h–i,** A close-up view for the representative cryo-EM densities of the already-existed disulfide bonds (h) and the firstly identified ones (i).

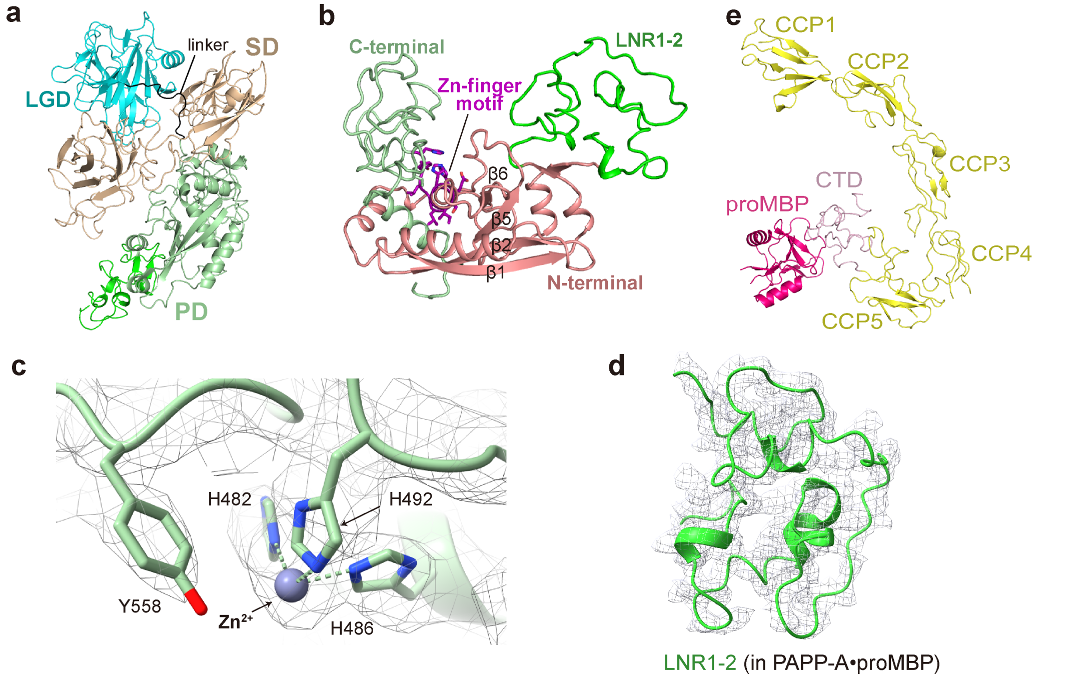

Supplementary Fig. S4 The overall structural compositions of PAPP-A.

a, LGD and PD locate at both sides of SD. **b,** Subdomains of PD consist of N-terminal (salmon), LNR1–2 (green), and C-terminal (palegreen). Catalytic zinc-finger motif ($H_{482}EIGHSLGLYH_{492}$) is highlighted in purple sticks. **c,** The mesh density for the catalytic residues and the coordinated Zn^{2+} ion. **d,** The density for LNR1–2 (residues 332 to 414) in PAPP-A•proMBP complex is well enough for accurate model building. **e,** The tandem CCP modules and CTD are flexible and the CTD solely interacts with proMBP.

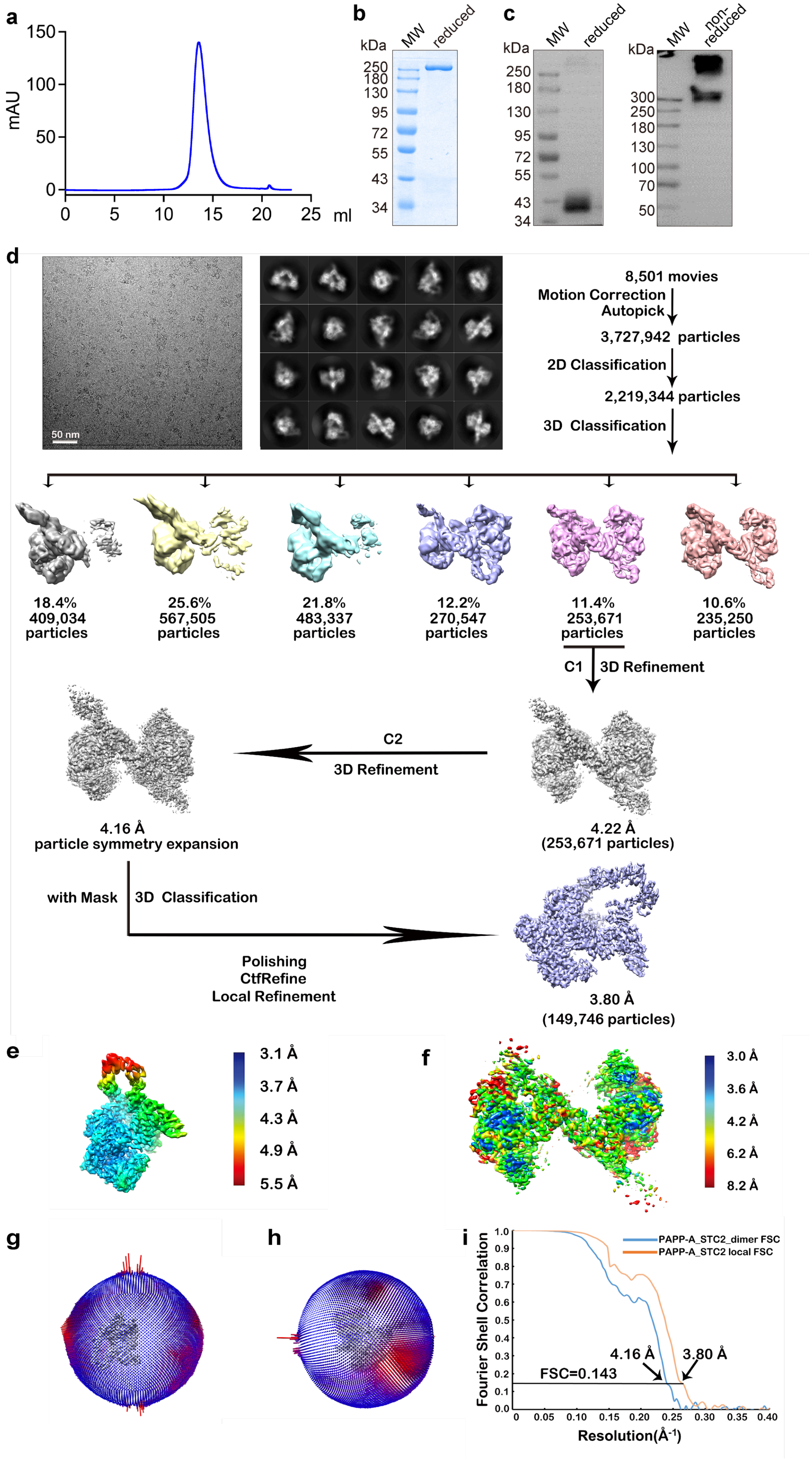
a
150
100
50
0
mAU
0 5 10 15 20 25 ml
b
kDa
MW
reduced
250
180
130
95
72
55
43
34
c
kDa
MW
reduced
250
180
130
95
72
55
43
34
kDa
MW
non-reduced
300
250
180
130
100
70
50
d
50 nm
8,501 movies
Motion Correction
Autopick
3,727,942 particles
2D Classification
2,219,344 particles
3D Classification
18.4%
409,034
particles
25.6%
567,505
particles
21.8%
483,337
particles
12.2%
270,547
particles
11.4%
253,671
particles
10.6%
235,250
particles
C1
3D Refinement
C2
3D Refinement
4.16 Å
particle symmetry expansion
4.22 Å
(253,671 particles)
with Mask
3D Classification
Polishing
CtfRefine
Local Refinement
3.80 Å
(149,746 particles)
e
3.1 Å
3.7 Å
4.3 Å
4.9 Å
5.5 Å
f
3.0 Å
3.6 Å
4.2 Å
6.2 Å
8.2 Å
g
h
i
PAPP-A_STC2_dimer FSC
PAPP-A_STC2 local FSC
Fourier Shell Correlation
4.16 Å
3.80 Å
FSC=0.143
0 0.05 0.10 0.15 0.20 0.25 0.30 0.35 0.40
Resolution(Å-1)

Supplementary Fig. S5 Purification, characterization, and cryo-EM structural analysis of the human PAPP-A•STC2 complex.

a, Size-exclusion chromatography elution profile. **b,** Gel electrophoresis of the PAPP-A•STC2 complex by Coomassie-blue stained reduced SDS-PAGE. **c,** Reduced (left) and non-reduced (right) western blots of the purified PAPP-A•STC2 complex using STC2 antibody. **d,** Workflow of cryo-EM data processing of the PAPP-A•STC2 complex. **e–f,** 3D density maps for the half (e) and dimer (f) colored according to local resolution (Å). **g–h,** The orientation distributions for the particles from the dataset of the half (g) and dimer (h). **i,** Gold-standard Fourier shell correlation (FSC) curves with estimated resolution at 0.143 of the final density map of dimeric PAPP-A•STC2 complex (blue) and local half (orange).

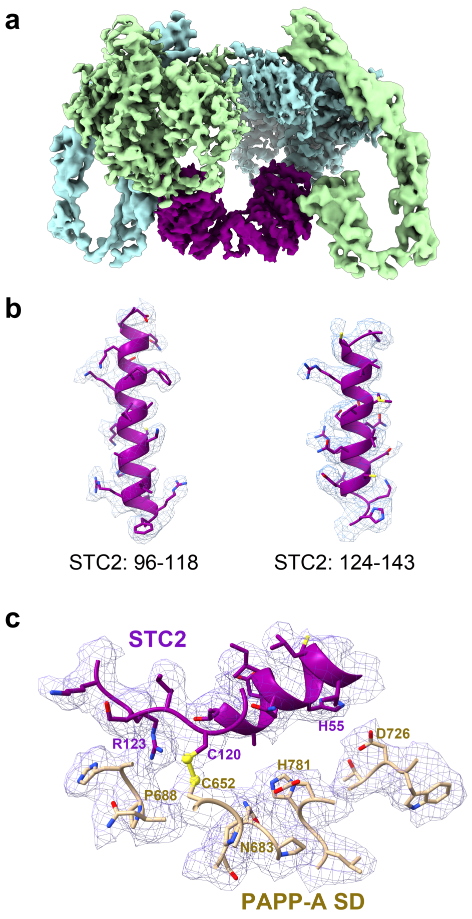

Supplementary Fig. S6 Local electron microscopy densities for the PAPP-A•STC2 complex.

a, The electron microscopy map for the PAPP-A•STC2 complex. **b,** A close-up view for the representative cryo-EM densities of STC2. **c,** The interface density between STC2 and SD.

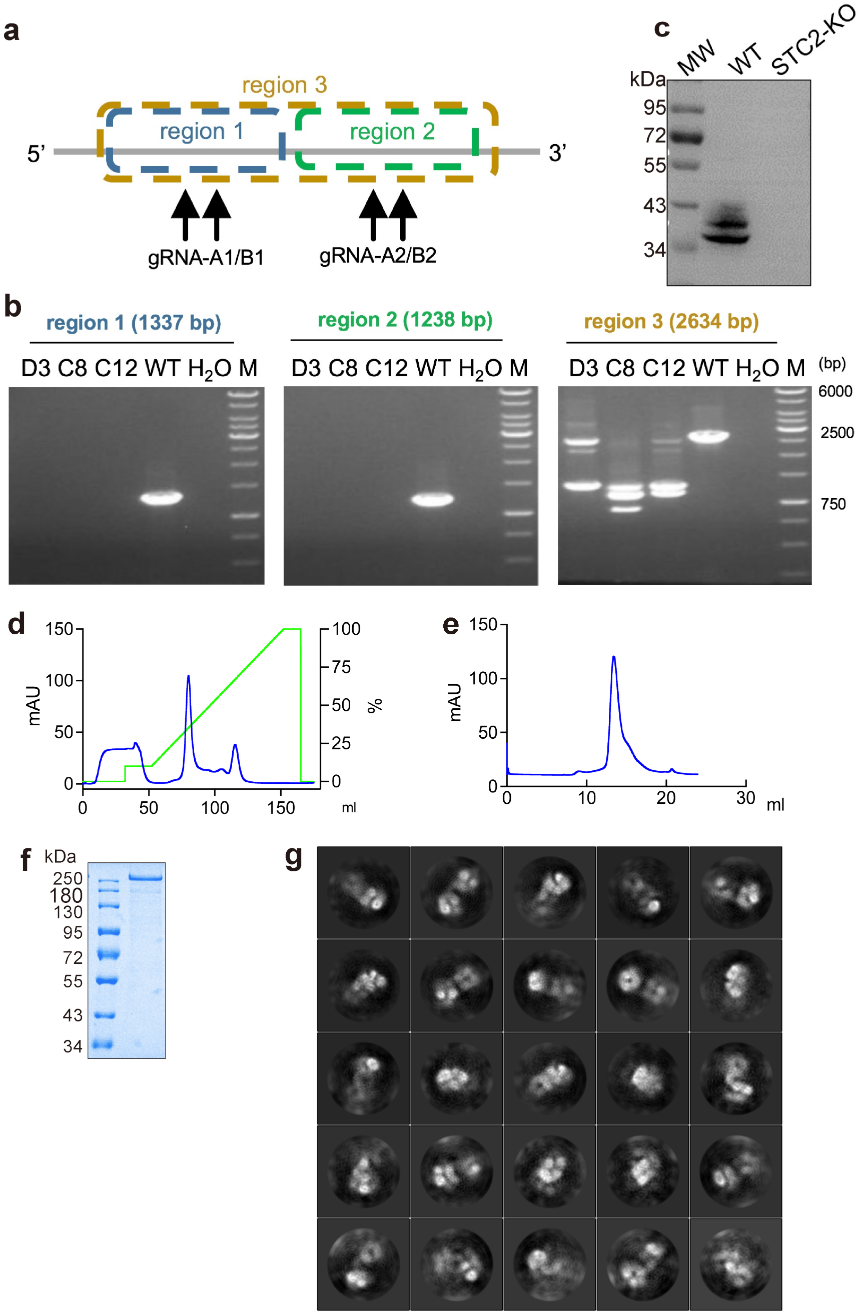

Supplementary Fig. S7 Construction of STC2-knockout HEK293T cell line, apo PAPP-A purifications and cryo-EM analyses. a, A diagram of gRNA design and targeting. **b,** PCR validation of single colonies identified C8 as a homozygous knock-out colony. **c,** Western blotting of the STC2-KO cells compared with wild-type (WT) HEK293T cells using STC2 antibody. **d–e,** Representative ion-exchange (d) and size-exclusion (e) chromatography of apo PAPP-A purification. **f,** Coomassie-blue staining of the apo PAPP-A on the reduced SDS-PAGE. **g,** Two-dimensional class averages of the apo structure.

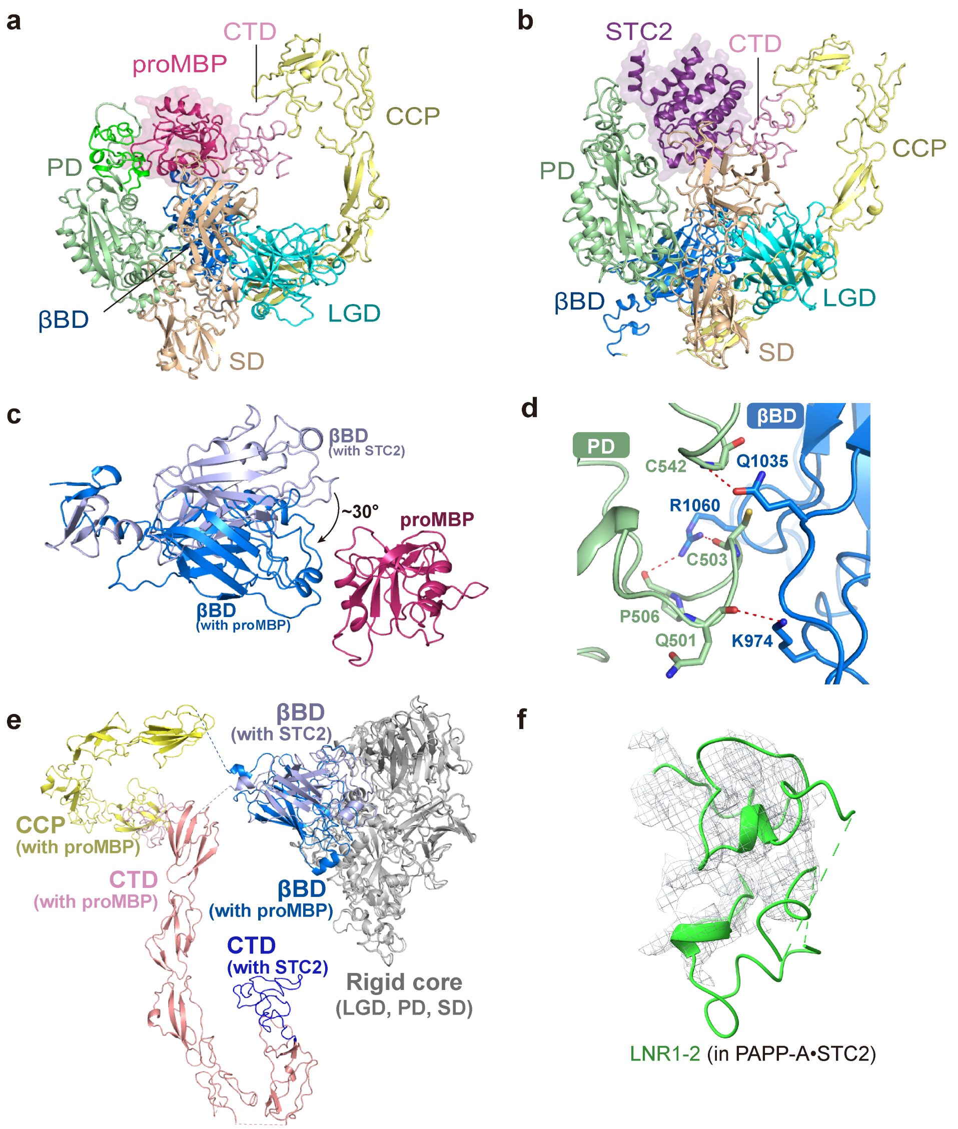

Supplementary Fig. S8 Structural comparisons between the two complexes. a–b, Domain colored structures of PAPP-A•proMBP (a) and PAPP-A•STC2 (b) complexes. For clarification, half of the PAPP-A dimer with LGD/PD/SD/βBD from one subunit and CCP/CTD from the other subunit are shown. ProMBP and STC2 are shown in both cartoon and surface. **c,** βBD rotates for approximately 30° in the presence of proMBP. **d,** Detailed interactions of PD and βBD in the PAPP-A•proMBP complex mediated by several hydrogen bonds. **e,** One copy of PAPP-A subunits from the two complexes are superimposed by aligning the rigid core (LGD, PD, and SD). Rigid core is uniformly colored gray while movable regions are differentially colored. **f,** The density for LNR1–2 in PAPP-A•STC2 complex is relatively weak when compared to that in PAPP-A•proMBP complex (Supplementary Fig. S4d).

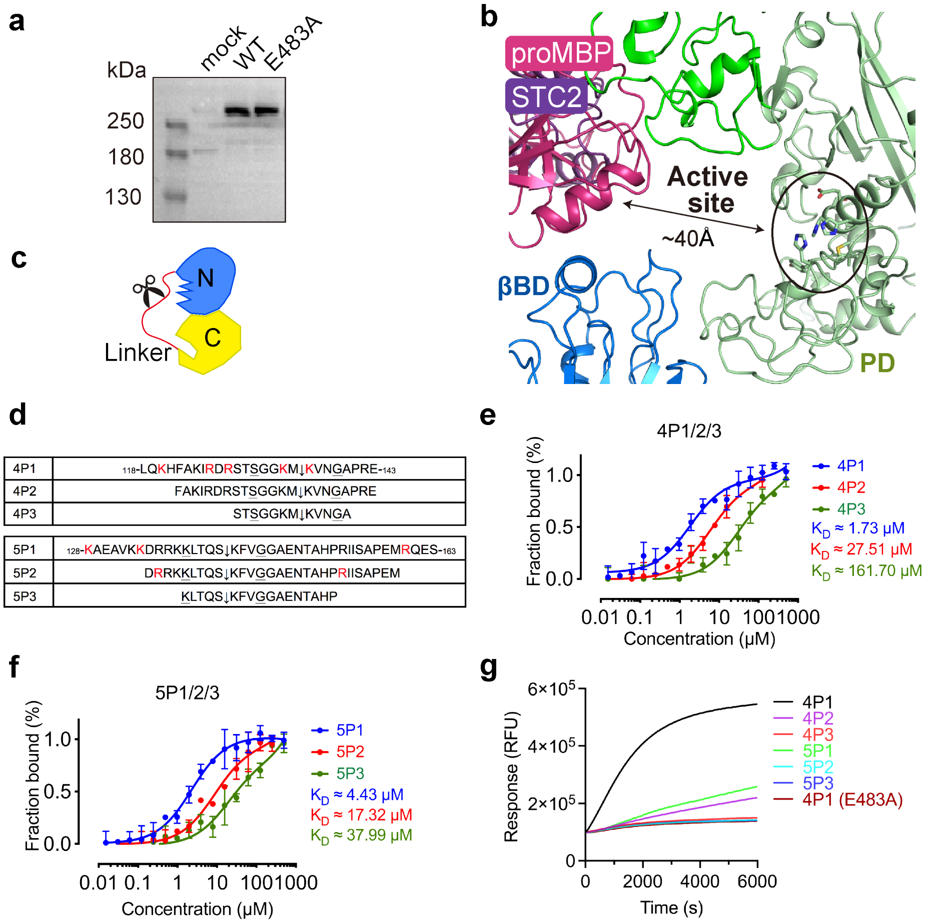

Supplementary Fig. S9 Cleavage properties of PAPP-A towards IGFBP-derived peptides.

a, Standardized amounts of PAPP-A enzyme were added in the catalytic reaction (detected by western blot using MBP-tag antibody). **b,** The binding sites of proMBP and STC2 are around 40 Å from the active site. **c,** The cartoon representation of IGFBPs. **d,** IGFBP4- and IGFBP5-derived peptides are designated 4P1, 4P2, and 4P3, and 5P1, 5P2, and 5P3, respectively. Basic residues are shown in red, arrows indicate cleavage sites, and underlined residues indicate substitution by Lys(Abz) or Tyr(NO_2) for the facilitation of FRET detection. The innocent mutation to Lys or Try was determined to possess the same K_D as the wild type (data not shown). **e–f,** MST analyses of PAPP-A binding properties of peptides derived from IGFBP4 (e) and IGFBP5 (f). **g,** FRET analysis of wild-type and inactive PAPP-A (E483A) cleavage on IGFPB4- and IGFBP5-derived peptides.

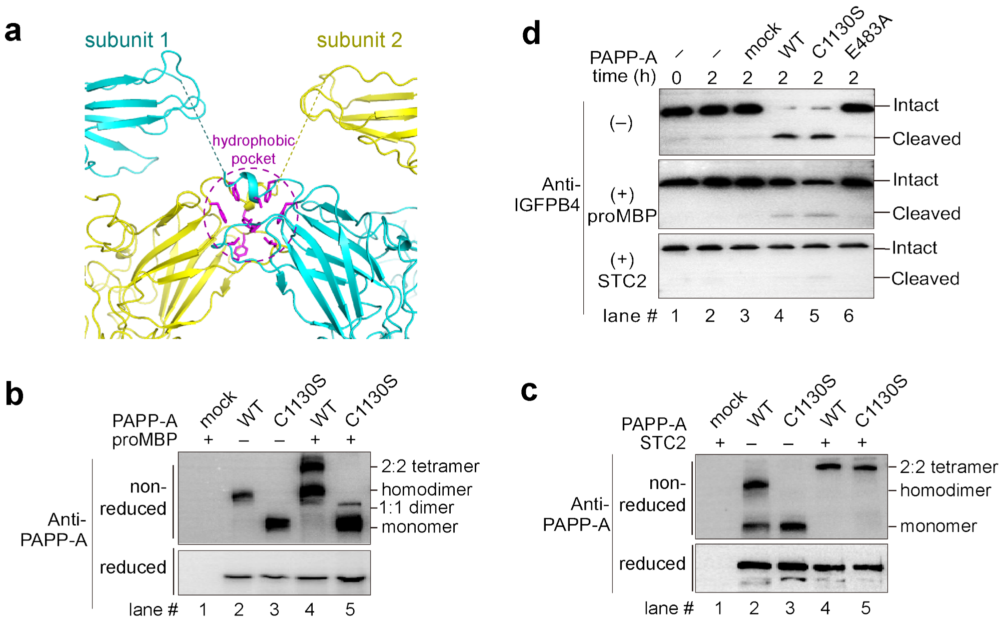

Supplementary Fig. S10 Modulators could inhibit the monomerized PAPP-A. a, A cartoon representation of the dimeric interface. The side chains of the hydrophobic pocket (formed by Val1095, Leu1152, Phe1156, Phe1181, Phe1184, and Val1187) are shown in magenta. **b,** Oligomerization state of proMBP with the monomerized PAPP-A (C1130S) or WT PAPP-A illustrated by both non-reduced and reduced electrophoresis. 2:2 tetramer, homodimer, 1:1 heterodimer, and monomer positions are indicated. **c,** Oligomerization state of STC2 and PAPP-A similar in b. **d,** The enzymatic activities of PAPP-A variants without modulator (top), with proMBP (middle) or with STC2 (bottom).

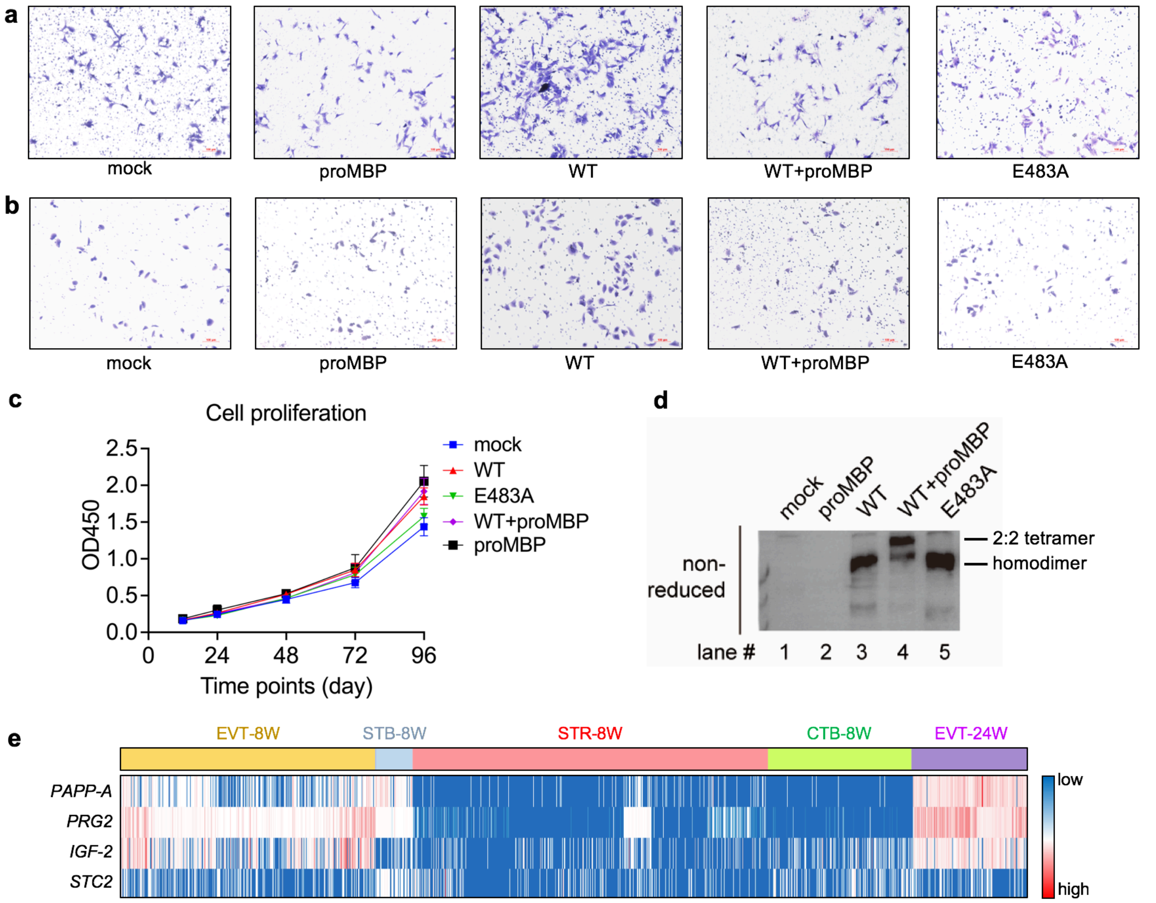

Supplementary Fig. S11 Functional identification of PAPP-A and proMBP in EVTs and their expressions in placenta.

a, Representative images of cell invasion assay using HTR8/SVneo cell lines with the transfection of the corresponding plasmids. Empty vector (mock) was transfected as control. WT, wild-type; E483A, mutation of Glu483 to Ala. **b,** Representative images of cell migration assay similar as a. **c,** Cell proliferation remained undifferentiated between these groups illustrated by the CCK-8 assay. **d,** PAPP-A and proMBP form complex when being co-expressed in HTR8/SVneo cells. **e,** Heatmap of the mRNA expression of *PAPP-A*, *PRG2*, *IGF-2*, and *STC2* from 8-week or 24-week placenta. Single-cell transcriptome data were taken from the published work[1].

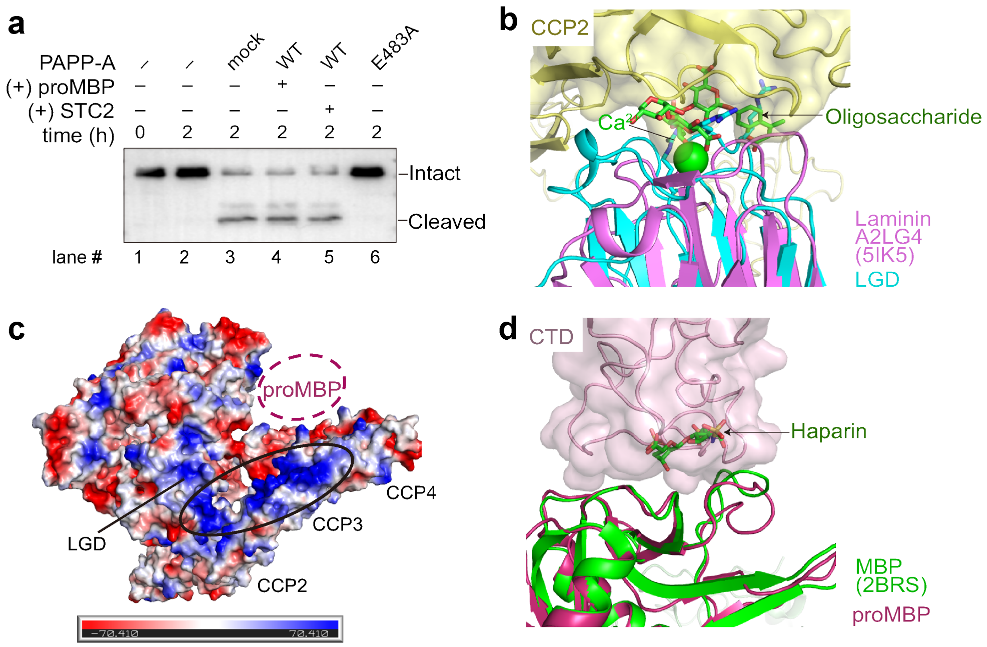

Supplementary Fig. S12 The assembly and disassembly of the PAPP-A complexes.

a, In the presence of purified proMBP protein or STC2 protein (+), the cleavage of IGFBP4 is not influenced. **b,** CCP2–LGD interface. Laminin A2LG4 (PDB code: 5IK5) is superimposed with LGD. A2LG4-bound oligosaccharide (shown in green sticks) clashes with CCP2 (shown in surface). Ca^{2+} ion is shown in green sphere. **c,** Vacuum electrostatics of PAPP-A in PAPP-A•proMBP complex. LGD, CCP2, CCP3, and CCP4 are indicated. Black oval highlights the highly basic region embedded in CCP3 and CCP4. **d,** CTD–proMBP interface. Matured form of proMBP (PDB code: 2BRS) is superimposed with proMBP. Matured MBP-bound heparin (shown in green sticks) clashes with CTD (shown in surface).

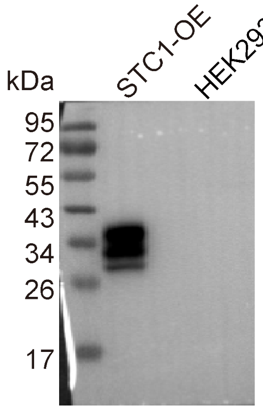

Supplementary Fig. S13 The expression of STC1 in HEK293F cells. The background expression of STC1 has not been detected in HEK293F cells. Recombinant STC1 overexpression (STC1-OE) was used as control.

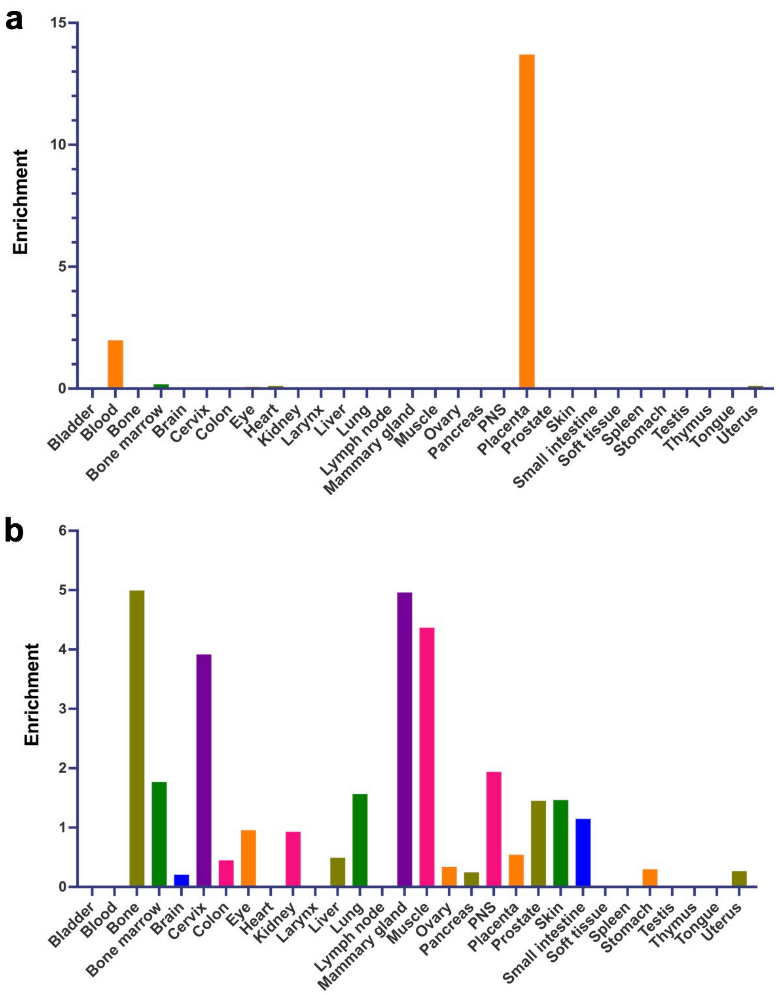

Supplementary Fig. S14 Relative expression levels of *PRG2* and *STC2* in various organs.

a, *PRG2* is extremely high-expressed in placenta and also expressed in blood. **b,** *STC2* is expressed broadly in different tissues. Data are obtained from TiGER database[2].

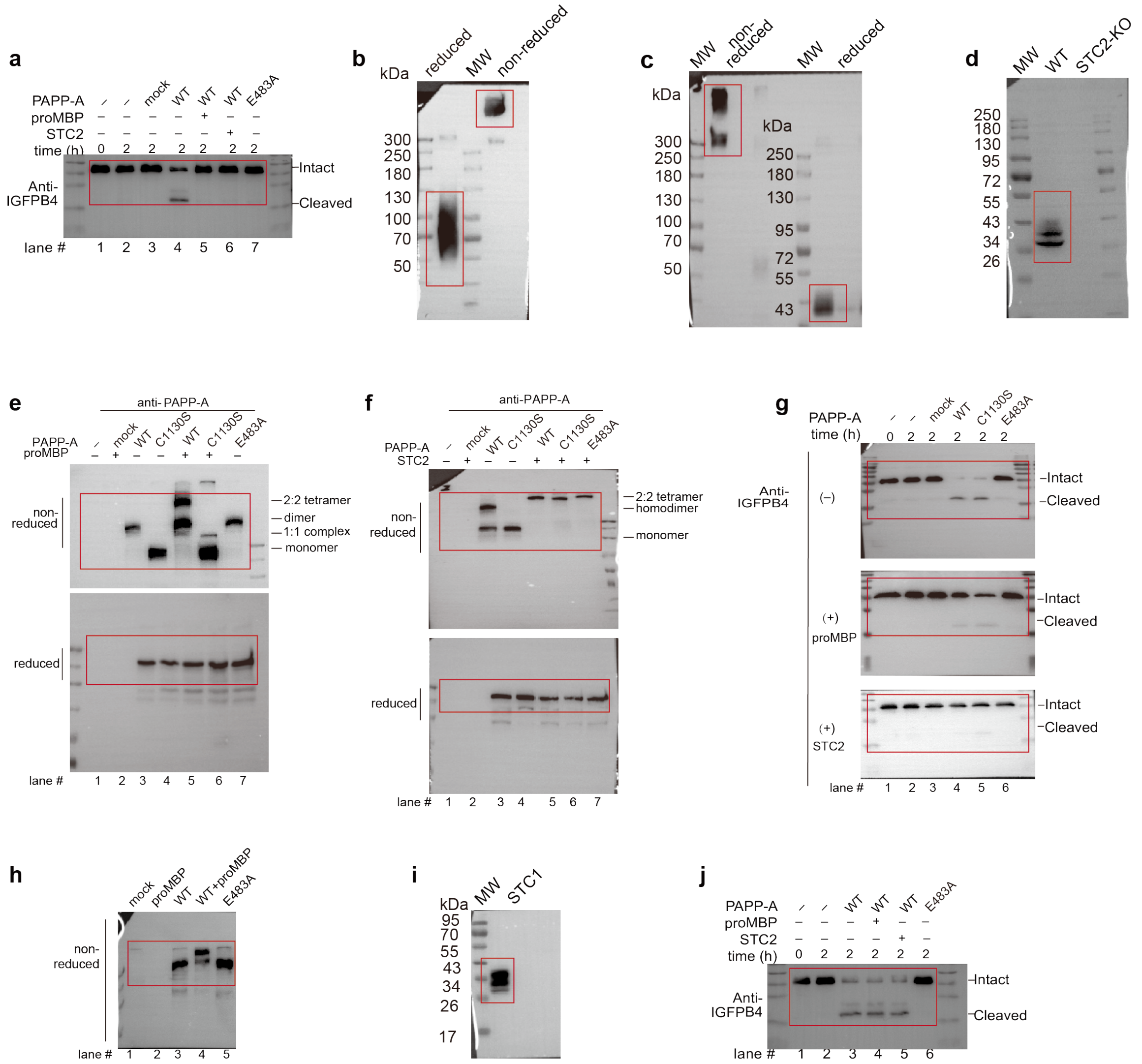

Supplementary Fig. S15 Uncropped gel images of all the western blots.

a, The blot for Fig. 5a. The red boxes indicate the presented bands in the main figures. **b,** The blots for Supplementary Fig. S1e. **c,** The blots for Supplementary Fig. S5c. **d,** The blot for Supplementary Fig. S7c. **e,** The blot for Supplementary Fig. S10b. **f,** The blots for Supplementary Fig. S10c. **g,** The blots for Supplementary Fig. S10d. **h,** The blots for Supplementary Fig. S11d. **i,** The blots for Supplementary Fig. S13. **j,** The blots for Supplementary Fig. S12a.

Supplementary Table S1. Cryo-EM data collection, refinement and validation.

	**2:2 PAPP-A• proMBP heterotetramer (EMD-34738; 8HGG)**	**1:1 PAPP-A• proMBP subcomplex (EMD-33621; PDB 7Y5N)**	**2:2 PAPP-A• STC2 heterotetramer (EMD-34739; 8HGH)**	**1:1 PAPP-A• STC2 subcomplex (EMD-33622; PDB 7Y5Q)**
**Data collection and processing**				
Voltage (kV)	300		300	
Microscope	FEI Titan Krios G3		FEI Titan Krios G3	
Camera	Gatan K2 Summit with energy filter		Gatan K2 Summit with energy filter	
Magnification (calibrated)	130,000×		130,000×	
Electron exposure (e⁻/Å²)	60.1		59.8	
Exposure rate	7.23 e⁻/Å²/s		7.19 e⁻/Å²/s	
Number of frames per micrograph	32		32	
Energy filter slit width	20 eV		20 eV	
Automation software	SerialEM		SerialEM	
Defocus range (μm)	-0.7 to -1.2		-0.7 to -1.2	
Pixel size (Å)	1.052		1.055	
Micrographs used	8,646		8,501	
Initial particle images (no.)	1,497,910		3,727,942	
Final particle images (no.)	261,371	142,469	253,671	149,746
Symmetry imposed	C2	C1	C2	C1
Map resolution (Å)	3.64	3.45	4.16	3.80
FSC threshold	0.143	0.143	0.143	0.143
**Refinement**				
Initial model used (PDB code)	1H8U	1H8U	–	–
Resolution (Å) at 0.143 FSC of masked reconstruction	3.64	3.45	4.16	3.80
Resolution (Å) at 0.5 FSC of masked reconstruction	4.08	3.91	4.58	4.10
Map sharpening B factor (Å²)	-157.102	-89.5997	-160	-157.413
Model composition				
Non-hydrogen atoms	25,406	13,035	25,496	12,754
Protein residues	3,238	1,639	3,266	1,634
Ligands	Zn:2	Zn:1<br>NAG:12<br>CA:7	Zn:2	Zn:1
R.m.s. deviations				
Bond lengths (Å)	0.003	0.004	0.003	0.004
Bond angles (°)	0.971	1.024	0.941	0.986
Validation				
MolProbity score	1.98	2.17	1.98	2.24
Clashscore	4.15	4.38	3.93	4.96
Poor rotamers (%)	4.05	4.78	5.22	5.87

Ramachandran plot				
Favored (%)	96.55	94.60	96.63	94.44
Allowed (%)	3.45	5.40	3.37	5.56
Disallowed (%)	0.00	0.00	0.00	0.00
C-beta outliers (%)	0.00	0.00	0.00	0.00
CaBLAM outliers (%)	2.44	3.15	2.27	3.05

Supplementary Table S2. Clinical characterization of sIUGR samples.

sIUGR Type	GDM	HDCP	TTTS	Gestational age at delivery (weeks)	Birthweight of the 1st fetus (grams)	Birthweight of the 2nd fetus (grams)
I	NO	NO	NO	35+6	2,540	1,900
I	YES	NO	NO	36	2,210	1,870
I	YES	NO	NO	36+1	2,090	2,810
I	NO	NO	NO	32	2,120	1,600
I	NO	NO	NO	35	2,350	1,730
I	NO	YES	NO	35+4	2,820	1,930
I	YES	NO	NO	36+4	2,710	2,340
I	NO	NO	NO	35	1,710	2,510
I	YES	NO	NO	34	2,280	1,740
I	NO	NO	NO	35	2,390	1,390
I	NO	YES	NO	34+1	2,340	1,290
I	NO	NO	NO	35	1,200	2,190

GDM, Gestational diabetes mellitus; HDCP, Hypertensive disorder complicated pregnancy; TTTS, twin-to-twin transfusion syndrome.

Supplementary Video 1. Architecture of PAPP-A•proMBP complex.
Overview of the PAPP-A•proMBP complex as well as zoom-in onto an proMBP subunit. All the domains in the 2:2 heterotetramer are indicated. Color scheme according to Fig. 1a.

Supplementary Video 2. Architecture of PAPP-A•STC2 complex.
Overview of the PAPP-A•STC2 complex as well as zoom-in onto STC2 dimer. All the domains in the 2:2 heterotetramer are indicated. Color scheme according to Fig. 3a.

Supplementary Video 3. The butterfly wing-flap of PAPP-A in two complexes.
Conformational twist of PAPP-A in PAPP-A•STC2 and PAPP-A•proMBP complexes looks like a "butterfly wing-flap". Structures are superimposed according to PAPP-A dimer. PAPP-A in PAPP-A•STC2 are colored yellow and PAPP-A in PAPP-A•proMBP are colored cyan.

References

1 Liu, Y. *et al.* Single-cell RNA-seq reveals the diversity of trophoblast subtypes and patterns of differentiation in the human placenta. *Cell Res* **28**, 819-832, doi:10.1038/s41422-018-0066-y (2018).

2 Liu, X., Yu, X., Zack, D. J., Zhu, H. & Qian, J. TiGER: a database for tissue-specific gene expression and regulation. *BMC Bioinformatics* **9**, 271, doi:10.1186/1471-2105-9-271 (2008).
